# Design and Synthesis of Peptide-Tagged Cubosome Nanocarriers for the Targeted Delivery of Paclitaxel in EGFR Overexpressing Breast Cancer

**DOI:** 10.1021/acsbiomaterials.5c02193

**Published:** 2026-02-19

**Authors:** Arindam Pramanik, Riya Rani, Bhavna Jha, Devlina Das Pramanik, Prashant Mishra

**Affiliations:** † Amity Institute of Biotechnology, 663345 Amity University, Noida 201301, India; ‡ Department of Biochemical Engineering and Biotechnology, Indian Institute of Technology Delhi, Hauz Khas, New Delhi 110016, India

**Keywords:** cubosomes, 3D tumor spheroids, epidermal growth factor receptor, peptide targeting, *in vivo* tumor model

## Abstract

Targeted delivery of chemotherapeutic agents can reduce systemic toxicity and enhance therapeutic outcomes by increasing the level of drug accumulation at tumor sites. In this study, we developed lipid-based cubosomal nanocarriers with an optimal size of 157 ± 20 nm for effective tumor penetration. This work represents the first demonstration of actively targeting cubosomes to epidermal growth factor receptors (EGFR) using a short peptide ligand. The peptide-functionalized cubosomes exhibited selective uptake of up to 75% in EGFR-overexpressing MDA-MB-468 breast cancer cells while showing minimal uptake (9%) in EGFR-negative HEK-293 cells. Paclitaxel-loaded targeted cubosomes significantly reduced MDA-MB-468 cell viability (47% survival at 60 μg/mL after 24 h) with negligible cytotoxicity in HEK-293 cells (87% survival). In 3D spheroid models, the survivability further decreased to 13% in MDA-MB-468 spheroids after 48 h, whereas HEK-293 spheroids remained largely unaffected. *In vivo*, targeted treatment suppressed tumor progression, yielding a mean tumor volume of 330 mm^3^, compared to 675 mm^3^ and 770 mm^3^ in untargeted and control groups, respectively, without observable liver or kidney toxicity. These results highlight the therapeutic potential of peptide-tagged cubosomes for the selective treatment of EGFR-expressing cancers.

## Graphical abstract

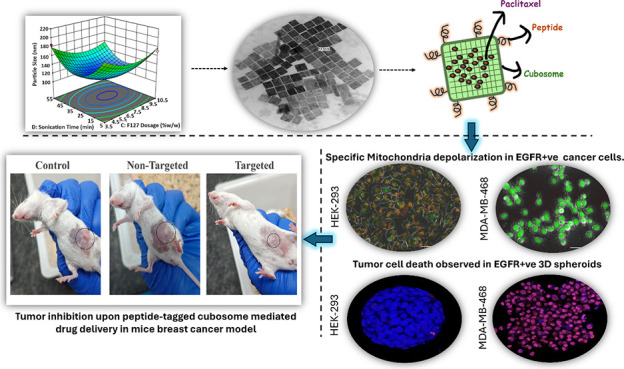

## Introduction

Lipid-based nanoparticles have emerged as one of the most successful nanocarrier platforms, surpassing many polymeric and metallic systems, with several formulations achieving clinical translation, including Doxil and Amphonex.^1^ An emerging class of lyotropic liquid crystalline lipidic nanoparticles also called ‘cubosomes’ has recently gained attention as nanotherapeutics including drug carriers for antimicrobial and cancer therapeutics.^2–5^ These have interior nanostructures with two- or three-dimensional periodicity, such as hexagonal or cubic symmetry, and are often stabilized by a polymer corona. Compared to conventional liposomes and other nanocarriers, cubosome nanoparticles offer several advantages such as porosity, structural versatility, improved stability, high encapsulation efficiency (due to their greater internal surface area), and biocompatibility as they consist of food-grade materials.^5^ Their amphiphilic nature allows them to encapsulate both hydrophobic and hydrophilic therapeutics such as nucleic acids (DNA^6^ and short interfering RNA^7^), imaging agents,^8^ and drug molecules.^9^ Cubosomes also offer a controlled release of drugs, such as in response to pH changes or facilitated cellular uptake. Several chemotherapeutic drug molecules such as paclitaxel,^9^ methotrexate,^10^ doxorubicin, and cisplatin^2^ have been encapsulated in cubosomes and studied for their efficacy in cancer cells. In our recent work, we have specifically delivered cubosomes in colorectal and breast cancer cells using carcinoembryonic antigens and CD44 receptors, respectively, using affimer proteins and hyaluronic acid as ligands.^11,12^

Aberrant EGFR overexpression drives oncogenesis by promoting uncontrolled growth, angiogenesis, and metastasis.^13^ EGFR-targeted therapies, such as tyrosine kinase inhibitors and monoclonal antibodies, have shown clinical benefits.^14^ The EGFR receptor has been widely utilized for targeting drugs to cancer cells by conjugating with cetuximab (EGFR binding monoclonal antibody).^15^ Additionally, a short 12 amino acid peptide sequence YHWYGYTPQNVI (GE11) has also been identified which has high specificity and binding affinity to the EGFR receptor.^16^ In this work, we present an interesting response surface methodology approach for optimizing cubosome size for application as a drug carrier. We further present a novel approach of using a short peptide as a targeting ligand for delivering cubosomes in EGFR expressing cancer cells. We have selected an analog of the GE11 peptide (with a single amino acid change) i.e. YHWYGYTPENVI. This mutant peptide has a 1.6–2.6 fold increased targeting efficacy for the EGFR receptor in EGFR +ve MDA-MB-468 cells compared to GE11.^17^ It is observed that the peptide-tagged cubosomes can successfully localize in EGFR expressing cells but not in EGFR negative cells. Upon paclitaxel encapsulation, there is a significant amount of apoptosis induced upon targeted delivery specifically in the EGFR+ve cells, as observed from both *in vitro* and *in vivo* studies. Thus, we here present a novel approach of utilizing a cost-effective short peptide unlike the high-cost monoclonal antibody (cetuximab) for targeted delivery of drugs through cubosome nanocarriers to enhance the therapeutic outcome in EGFR +ve cancers.

## Materials and Methods

### Synthesis of Cubosomes, Drug Encapsulation, and Peptide Tagging

Glyceryl monooleate (GMO), DSPE-PEG-2000 amine (DSPE), Pluronic F-127, and paclitaxel were purchased from Merck, Germany. Cubosomes were synthesized using various ratios of GMO (80–95% w/w) and DSPE (5–10% w/w) codissolved in chloroform (Merck, Germany) and mixing them followed by drying under inert conditions under N_2_ gas. To confirm the complete removal of the solvent (chloroform), the sample was left in a desiccator overnight at room temperature. Next, Pluronic F-127 (F127) was dissolved in sterile phosphate buffer saline (PBS; Sigma-Aldrich, USA) and added to the dried lipid film of GMO–DSPE. The concentration of F127 was varied between 3.5 and 10% (w/w) GMO to optimize the size. Cubosomes were synthesized by sonicating the mixture in ice cold conditions using a Q125 sonicator (Qsonica, USA) for varying time points (5–55 min) in pulse mode (1 s pulse, 1 s gap) at 65% amplitude. For drug loaded cubosomes, paclitaxel (Pxl; Merck, USA) was dissolved in ethanol and added in various amounts (1–7.5% w/w) to the codissolved lipid mixtures before drying under N_2_ gas. The same steps described above for cubosome synthesis were then followed. The drug loaded cubosomes were placed in Slide-A-Lyzer cassettes (3.5K MWCO, Thermo Fisher Scientific, USA) in PBS at 25 °C to remove any unencapsulated drug with PBS as an external buffer. For analyzing cubosome localization *in vitro*, Tetramethyl rhodamine isothiocyanate (TRITC Thermo Fisher Scientific, USA) 0.5% w/w of GMO was codissolved with the lipid mixtures before drying with N_2_ gas. Similar to paclitaxel, TRITC labeled cubosomes were dialyzed in dialysis cassettes to remove any free dye. For peptide conjugation, 1 mM of peptide was dissolved in Milli-Q water, and 0.5 mM of 1-ethyl-3-(3-(dimethylamino)­propyl)­carbodiimide (EDC) and 0.5 mM of *N*-hydroxysuccinimide (NHS) were added to activate the terminal carboxylic group. The reaction was continued for 18 h at 25 °C. Next, the reaction mixture was added to the cubosomes to form an amide bond between the carboxylic group of the peptide and the amine group of DSPE in the cubosomes. The unconjugated peptide was removed by dialysis.

### Optimization – Box Behnken Design

A Box–Behnken design was utilized to evaluate the effects of various parameters and to identify significant single factors, interactions, and quadratic terms using Design Expert software (version 13). Each factor was tested at three levels: −1, 0, and +1, representing low, medium, and high values, respectively. The design included 29 runs and focused on four factors: A (GMO dosage%w/w), B (DSPE dosage (%w/w)), C (F127 dosage (%w/w)), and D (sonication time in minutes). The parameter ranges were selected based on preliminary experiments conducted prior to optimization and are represented in Table 1.

**Table 1:** Selected Range of Parameters for Performing Response Surface Analyses

			Range Represented as Coded Value^a^		
S. No.	Factor	Unit	–1	0	+1
1	A: GMO Dosage	% w/w	90.5	95	99.5
2	B: DSPE Dosage	% w/w	9.5	5	0.5
3	C: F127 Dosage	% w/w	3.5	7	10.5
4	D: Sonication Time	min	5	30	55

a Coded values −1, 0, and +1 represented low, medium, and high values for respective factors.

### Determination of Encapsulation Efficiency

Paclitaxel (Pxl), which is insoluble in water, was dissolved in anhydrous ethanol, and 5% w/w of Pxl was added to the lipid mixture of GMO:DSPE before drying with N_2_ gas. The cubosomes were synthesized as described above. Post cubosome synthesis, the unencapsulated drug was removed using the dialysis method. Then the cubosomes were dissolved in a 1:2 ratio of an ethanol:chloroform solution which was analyzed with a UV–Vis spectrophotometer (Shimadzu, Japan) at 231 nm for paclitaxel absorbance to determine the amount of paclitaxel encapsulated in the cubosomes. The encapsulation efficiency (EE%) for paclitaxel was calculated using eq 1

$$\mathrm{Encapsulation Efficiency}\;\left(\mathrm{EE}\%\right)=\frac{C_{e}}{C_{i}}\times 100$$ 1

where *C_e_* is the weight of Pxl encapsulated in the cubosomes, and *C_i_* is the weight of Pxl added.

### Size and Zeta Potential Determination

The hydrodynamic diameters of particles were measured by using Zetasizer Nano ZS90, Malvern, USA. For the measurement, the refractive index for the cubosome was set at 1.46 (with respect to GMO). The refractive index for PBS (dispersant) was set at 1.332, and the viscosity was set at 0.9053 cP. The sizes of bare cubosomes (Cbs) and peptide-tagged cubosomes loaded with Pxl (Cbs-Pxl-Pep) were measured by adding 10 μL of cubosomes into 990 μL of PBS in a quartz cuvette. The backscattering angle was set to 173°. The instrument equilibration time was set for 2 min at 25 °C, and samples were measured by running 20 cycles with each cycle having 10 runs. For surface charge, zeta-potential measurement was performed by adding 50 μL of Cbs-Pxl-Pep in 950 μL of deionized Milli-Q water (with a resistivity of 18.2 MΩ·cm at 25 °C) in a disposable zeta cuvette, post sample equilibrated for 2 min at 25 °C. The run parameter was set to 20 cycles with 10 runs in each cycle.

### Morphological Analysis of Cubosomes by High-Resolution Transmission Electron Microscopy (HR-TEM)

Morphological analysis of the cubosomes was done by using a high-resolution transmission electron microscope (Tecnai G^2^ 20 U TWIN) fitted with detectors BF/DF, CCD, and corrected optics. For this analysis, a 200 mesh carbon film coated on a nickel grid (Sigma-Aldrich, USA) was used. Ten μL of an 80 mg/mL concentration of Cbs-Pxl-Pep was added to the grid and left to air-dry at 24 °C in a desiccator overnight. The sample was imaged at 10000× magnification at an accelerating voltage of 200 kV. The image was captured using a CCD camera. Images were further analyzed using Fiji ImageJ software (NIH, USA).

### Cell Culture

Cancer cell lines MDA-MB-468, MDA-MB-231, MCF-7, and 4T1 and noncancerous HEK-293 were originally obtained from ATCC. Cells were maintained in Dulbecco’s Modified Eagle Medium (DMEM; Thermo Fisher Scientific, USA) with 10% fetal calf serum (FCS; Thermo Fisher Scientific, USA) and 5% penicillin/streptomycin (Thermo Fisher Scientific, USA). Cells were cultured in a humidified incubator with 5% CO_2_ and 37 °C.

### EGFR Expression Analysis and Cubosome Localization Study

MDA-MB-468 and HEK-293 cells were cultured on a glass coated chamber slide (Thermo Fisher Scientific, USA) in DMEM media with 10% FCS. After 48 h, the cell media was discarded, and cells were washed with PBS and fixed with 4% (w/v) paraformaldehyde (PFD; Sigma-Aldrich, USA) for 15 min at 24 °C. Cells were then washed with PBS to remove any residual PFD. Next, cells were permeabilized with 0.2% (v/v) Triton X-100 (Sigma-Aldrich, USA) in PBS at 4 °C for 15 min as permeabilization allows the antibody to bind to the antigens. Post permeabilization, cells were washed 5 times with PBS and blocked with 5% (v/v) FCS in PBS for 1 h at 4 °C. Cells were then incubated with antihuman EGFR mouse IgG1 monoclonal antibody (Thermo Fisher Scientific, USA) at a dilution of 1:1500 overnight at 4 °C. Further cells were incubated with AlexaFluor 488 labeled goat antimouse IgG antibodies (catalogue A-11001, Thermo Fisher Scientific, USA) at 1 μg/mL for 1 h in the dark at 24 °C. Next cells were washed with the wash buffer several times and mounted with Hoechst 33342 (Thermo Fisher Scientific, USA). Then cells were analyzed using confocal microscopy (TCS SP8, Leica Microsystems, Germany). Images were captured using a 100× magnification and further analyzed using Fiji ImageJ software (NIH, USA).

For cubosome localization studies, MDA-MB-468 and HEK-293 cells were grown in a glass coated chamber slide (Corning, USA) for 18 h under the optimum growth conditions. Cells were then treated with 25 μg/mL TRITC loaded cubosomes (Cbs) with and without peptide conjugation. Post incubation with the fluorescent cubosomes, cells were washed with PBS to remove any external nonspecifically attached cubosomes and further stained with (5 μg/mL) Hoechst 33342 for 15 min. Next, cells were imaged using a confocal microscope at 100× magnification.

### Cell Survival, Mitochondrial Membrane Potential, and Apoptosis Assays in 2D Culture

MDA-MB-468 and HEK-293 cells were seeded in 96-well tissue culture plates in complete growth media at densities of 1 × 10^4^ cells/well and incubated at 37 °C for 18 h. Next, cells were treated at varying concentrations (0–100 μg/mL) for 12 and 24 h with paclitaxel loaded cubosomes (Cbs-Pxl) and Cbs-Pxl-Pep. Post treatment, cells were incubated with MTT dye (Sigma-Aldrich, USA) for 3 h, and then the formazan crystals were dissolved in isopropanol (Sigma-Aldrich, USA) to measure the absorbance at 595 nm.^18^ Additionally, to confirm cell death from morphology, MDA-MB-468 and HEK-293 cells were seeded in a 24-well plate at a density of 5 × 10^4^ cells/well and treated with an IC_50_ dose of Cbs-Pxl-Pep for 12 and 24 h. Post treatment, cells were incubated with 5 μg/mL Hoechst 33342 and analyzed under a fluorescence microscope (Nikon, Japan).

For measuring the mitochondrial membrane potential, 5 × 10^4^ cells/well of MDA-MB-468 and HEK-293 were seeded in a 24-well plate. Then cells were treated with an IC_50_ dose of Cbs-Pxl-Pep for 12 and 24 h. Cells were then gently washed with PBS, incubated with 5 μM JC-1 dye (Thermo Fisher Scientific, USA) for 30 min at 37 °C, and then analyzed under a fluorescence microscope.

Flow cytometry (FACS) was used to analyze apoptosis using annexin V/propidium iodide. Briefly, MDA-MB-468 and HEK-293 cells were treated with an IC_50_ dose of Cbs-Pxl-Pep for 12 and 24 h. Post treatment, cells were washed with annexin binding buffer, then incubated with 2 μg/mL annexin V-FITC (Thermo Fisher Scientific, USA), and incubated for 15 min under dark conditions at 37 °C. Next, cells were washed with PBS, and 1 μg/mL propidium iodide (Thermo Fisher Scientific, USA) was added before FACS analysis (BD Bioscience, USA).

### 3D Spheroid Assay

3D Spheroids of MDA-MB-468 and HEK-293 were grown in low adherent round-bottom 96-well plates (Corning, USA) as reported in our previous work.^4^ Briefly, 2000 cells per well were added in each well containing complete medium with a 2.5% Matrigel matrix (Corning, USA) and centrifuged at 360 x g*g* for 15 min. Cells were then incubated for 48 h at 37 °C in the incubator for the 3D spheroids to form. The spheroids were then treated with the IC_50_ dose of Cbs-Pxl-Pep for 24 and 48 h. Post treatment the spheroids were stained with 5 μg/mL Hoechst 33342 (nuclear staining) and 2 μg/mL propidium iodide (dead cells staining).

For analyzing apoptosis in spheroids, a Western blot was performed to analyze the caspase 3 protein marker as reported earlier.^4^ Briefly, spheroids were treated with an IC_50_ dose of Cbs-Pxl-Pep for 48 h. Post treatment, spheroids were gently washed with PBS and incubated with lysis buffer with mild vortexing. The protein was collected as the standard protocol, and a Western blot assay was performed (BioRad, USA). The total protein was analyzed for caspase 3 and β-actin protein marker antibodies (Cell Signaling Technologies, USA).

### Animal Experiments

Female BALB/c female mice, aged 6 weeks, each weighing approximately 25 g, were used for *in vivo* studies. Mice were sourced from Rodent Research India Lab, Haryana. All experiments were performed following ethical guidelines of the Animal Ethical Committee (IAEC) of Amity University, Noida with a clearance certificate (CPCSEA/IAEC/AIP/2024/10/8). Mice were housed in individually ventilated cages with a 12 h day/night cycle, with provisions for *ad libitum* food and water. At the end of the experiment, mice were euthanized following carbon dioxide (CO_2_) asphyxiation, as per the guidelines of IAEC.

### *Ex Vivo* Hemolysis Study

Blood was collected from four female BALB/c mice via the retro-orbital plexus into K3-EDTA-coated collection tubes. Whole blood was centrifuged at 500 × g for 5 min at 4 °C to separate the red blood cells (RBCs), and the plasma was subsequently discarded. The RBCs were gently resuspended in a 150 mM NaCl solution, followed by repeated washing and dilution to a 1:50 ratio in PBS. For the controls, 1% (v/v) Triton X-100 was used as a positive control (100% lysis), whereas cells incubated in PBS served as the negative control (0% lysis). Triplicate samples of 200 μL of RBC suspension were treated with Cbs-Pxl or Cbs-Pxl-Pep at concentrations of 25, 50, or 100 μg/mL and incubated on an orbital shaker at 150 rpm for 1 h at 37 °C. After incubation, samples were centrifuged at 500 × g for 5 min to pellet intact cells, and the supernatants containing lysed RBCs were collected. Hemolysis was quantified by measuring the absorbance at 540 nm using a spectrophotometer.

### Tumor Targeting *in Vivo* Study

Female BALB/c mice were injected with 5 × 10^5^ 4T1 cells subcutaneously in the mammary fat pad. After 2 weeks, a tumor of around 150 mm^3^ was observed. Eighteen tumor bearing mice were randomly divided into 3 groups each having 6 mice. Group 1 (control) received an injection of 100 μL saline in the tail vein (intravenous). Group 2 received 100 μL of Cbs-Pxl (25 mg/kg of body weight) which served as the nontargeted group, whereas group 3 received 100 μL of Cbs-Pxl-Pep (25 mg/kg of body weight), serving as the targeted group. The administration was repeated three times, first dose on day 1, second dose on day 3, and final dose on day 5. Tumor volume and body weight of mice were monitored throughout the duration of the experiment. The experiment was terminated on day 24, and mice were euthanized using CO_2_ asphyxiation. Tumors along with vital organs were collected for toxicology studies using H&E staining.

## Results and Discussion

### Optimization of Parameters Using Box–Behnken Design

Various cubosomes have been formulated based on glyceryl monooleate (GMO) and phytantriol which have been extensively used for drug delivery applications.^19–21^ We here used GMO based cubosomes as reported in our recent research work.^3,4^ Cubosomes were made using the various combinations of GMO, DSPE-PEG2000-amine, and stabilized by pluronic F-127. Here DSPE-PEG2000-amine served as an additional stabilizer, but more importantly, it enabled tagging of the peptide ligand using amide bond coupling. The size of nanoparticles plays a crucial role in therapeutic efficacy. It has been reported that smaller nanoparticles <30 nm have low retention in the circulation and are rapidly eliminated from the body, whereas larger particles (>200 nm) have low penetration ability in the tumor vasculature and thus have low therapeutic efficacy.^22^ So, to achieve an optimum size range (100–200 nm), various combinations of GMO, DSPE, and F127 were used along with sonication time.

Response surface methodology (RSM) has been employed as a statistical approach for the identification of complex quantitative relations between parameters and responses.^5^ In our proposed research, a Box-Behnken design was employed to study the effects of four parameters, namely, GMO dosage (factor A), DSPE (factor B), F127 dosage (factor C), and sonication time (factor D). The responses from the respective experimental runs have been recorded in Table 2. The range for respective dosage values and sonication time has been considered based on our previous research on cubosome synthesis and its applicability.^3^ The optimum conditions were noted as follows: factor A (GMO dosage)- 95% w/w, factor B (DSPE dosage)- 5% w/w, factor C (F127 dosage)- 7% w/w, and factor D (sonication time)- 30 min. Under the mentioned optimum conditions, the particle size of the synthesized cubosomes was noted to be 110 ± 10 nm as shown in the transmission microscopy image (Figure 1B). Among several interaction factors, the interaction between stabilized F127 dosage (factor C) and sonication time (factor D) was found to be most significantly affecting the particle size (Figure 1A, 1C). However, the range of variations for the respective factors could be noted from the interaction plots. The interactions between the various single factors were represented as respective contour and 3D mesh diagram plots (Figures 1, S1–S2). Contour plots showed prominent ‘blue’ regions and shorter zones of “green”, “yellow”, and ‘red’ tinge. This suggested the dependency of the cubosomes on the respective interactions. With respect to factor A (GMO dosage), a prominent ‘blue’ zone was noted at a narrow dosage range of 92.75 (% w/w)–97.25 (% w/w) (Figure S1A-C). However, interactions between the DSPE dosage with F127 and sonication time revealed that the range of the DSPE dosage required for the size restriction of cubosomes was noted to be broader than the GMO dosage (Figure S1D-E).

**Figure 1:**
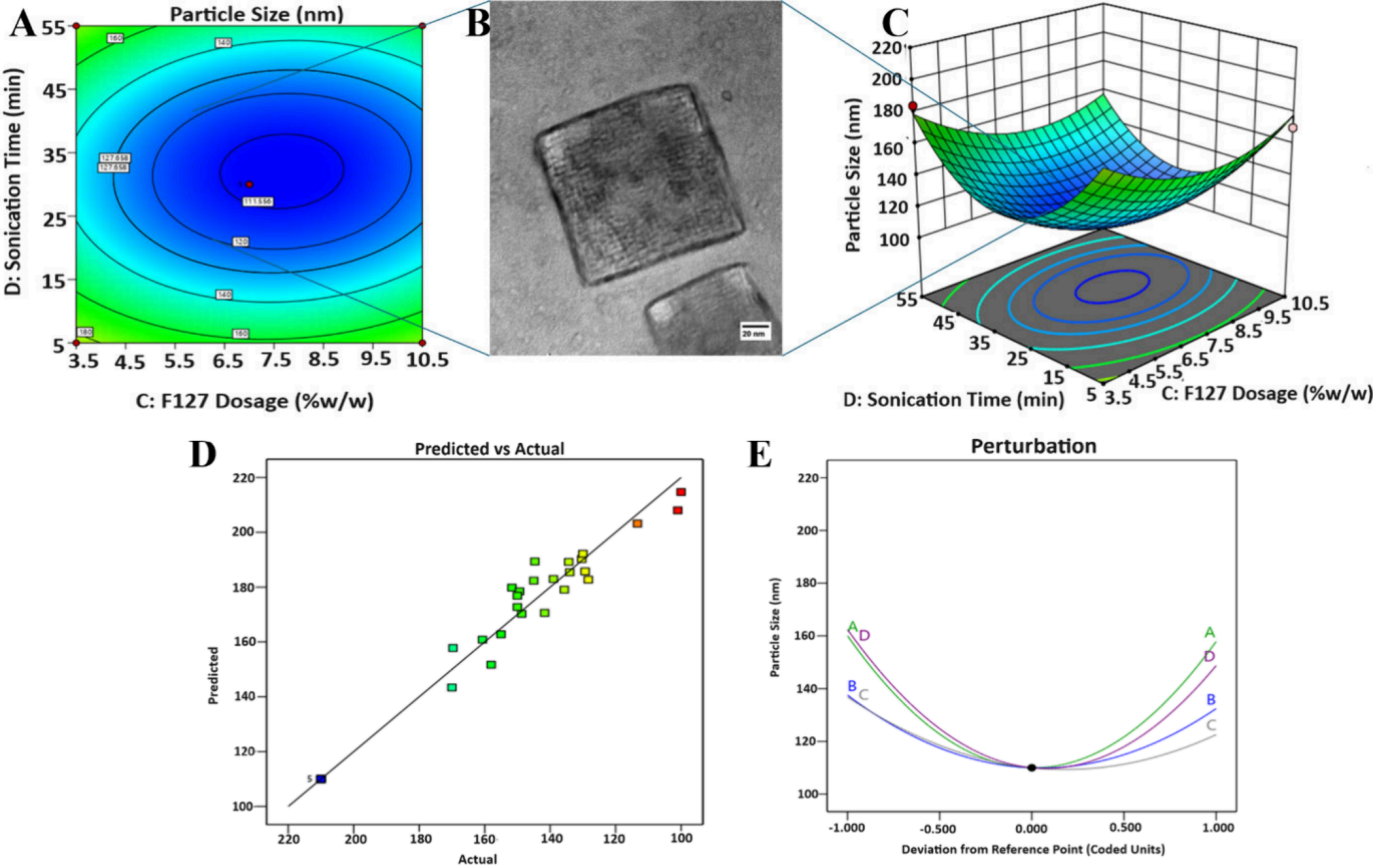
Interaction effects between significant single factors (C: F127 dosage; D: Sonication time) on cubosome particle size (A) contour plot; (B) transmission electron microscopy image of cubosome showing a 110 nm diameter as synthesized under optimal conditions [Optimal Conditions-Factor A (GMO Dosage): 95% w/w; Factor B (DSPE Dosage): 5% w/w; Factor C (F127 Dosage): 7% w/w; Factor D (Sonication time): 30 min]; (C) 3D mesh diagram; (D) predicted vs actual plot; (E) perturbation plot.

**Table 2:** Experimental Run Table Showing the Effects of Dosages and Sonication Time on the Particle Size of the Synthesized Cubosome^a^ ^,^ ^b^

***Run***	*Factor 1* **A: GMO Dosage** **(% w/w)**	*Factor 2* **B: DSPE Dosage** **(% w/w)**	*Factor 3* **C: F127 Dosage** **(%w/w)**	*Factor 4* **D: Sonication Time** **(min)**	*Response* **Particle Size**_**Exp**_ ^c^ **(nm)**	*Predicted Response* **Particle Size**_**Pred**_ ^d^ **(nm)**
1	95	9.5	3.5	30	159.3 ± 3.1	168.9
2	90.5	0.5	7	30	189.7 ± 2.5	190.2
3	95	0.5	7	5	175.3 ± 5.7	189.4
4	90.5	5	7	55	206.7 ± 11.5	203.2
5	95	5	7	30	110.0 ± 0.0	110
6	95	9.5	7	5	186.0 ± 5.3	185.4
7	99.5	5	3.5	30	175.0 ± 5.0	182.4
8	95	9.5	7	55	178.3 ± 12.6	170.6
9	90.5	5	7	5	219.0 ± 6.6	208
10	95	0.5	10.5	30	162.0 ± 7.2	151.7
11	90.5	9.5	7	30	168.3 ± 7.6	179.8
12	95	9.5	10.5	30	150.0 ± 5.0	143.4
13	99.5	9.5	7	30	181.0 ± 5.6	182.9
14	99.5	5	10.5	30	170.0 ± 5.0	172.8
15	99.5	0.5	7	30	191.7 ± 7.6	182.7
16	95	5	7	30	110.0 ± 5.8	110
17	95	5	10.5	5	170.7 ± 9.0	178.4
18	99.5	5	7	5	220.0 ± 10.0	214.7
19	99.5	5	7	55	190.0 ± 10.0	192.2
20	95	5	7	30	110.0 ± 2.3	110
21	95	5	3.5	55	184.3 ± 7.4	179.1
22	95	5	7	30	110.0 ± 2.3	110
23	95	5	7	30	110.0 ± 3.1	110
24	95	0.5	3.5	30	165.0 ± 5.0	162.8
25	90.5	5	10.5	30	171.3 ± 3.2	170.3
26	95	5	3.5	5	190.7 ± 4.0	185.7
27	95	0.5	7	55	170.0 ± 10.0	176.9
28	90.5	5	3.5	30	185.7 ± 4.0	189.2
29	95	5	10.5	55	150.3 ± 4.5	157.8

a Predicted values have been obtained from the point predictions suggested by the model.

b Average percentage error value was noted to be 3.46%

c Particle Size_Exp_: Particle size experimentally determined using dynamic light scattering (DLS).

d Particle Size_Pred_: Point prediction for cubosome particle size.

In addition to the contour plots, the effect of interactions on cubosome particle size was better represented as 3D mesh diagrams (Figure S2A-E). The plots revealed that the size of the cubosomes was quite dependent on the central values of the respective parameters. A deviation from the central values resulted in an increase in the cubosome size. As a vital additional inference with regard to the contour plot, we noted the significance of the central values for interactions involving GMO dosage (interaction factors: AB, AC, and AD) (Figure S2A-C). On the contrary, the variations in the size of cubosomes with respect to the central value were relatively lower for interactions involving DSPE dosage (interaction factors: BC, BD) (Figure S2D-E). The predicted versus actual plot showed a lower scatter of the data, which justified the model significance (Figure 1D). Deviations around the central values were depicted by the perturbation plot (Figure 1E).

Table S1 shows the analyses of variance for the respective single, interaction, and quadratic factors. The model significance was represented by the high F-value of 24.51 (p-value <0.0001). Respective p-values for factor C (p-value: 0.0140, F-value: 7.88) and factor D (p-value: 0.0177, F-value: 7.22) justified the significance of the factors in the size determination of cubosomes.

However, none of the interaction factors were noted to significantly affect the size of cubosomes. The correlation coefficient (R^2^) value and adequate precision value were noted to be 0.9608 and 16.5240, respectively, which justified the model significance. The coded eqs 2–3 as suggested by the software are shown below:

2 $$Particle\;Size\;\left(nm\right)=110-1.08A-2.56B-7.14C-6.83D+2.67AB+2.33AC-4.42AD-1.58BC-0.58BD-3.50CD+48.94A^{2}+24.99B^{2}+19.69C^{2}+45.57D^{2}$$

Based on the significant factors, the revised equation can be written as follows:

3 $$Particle\;Size\;\left(nm\right)=110-7.14C-6.83D+48.94A^{2}+24.99B^{2}+19.69C^{2}+45.57D^{2}$$

Based on the equations, the predicted values were calculated and are represented in Table 1. The average percentage error was noted to be less than 5% (3.465%).

Similar to our study, CTAB-modified *Polygonatum sibiricum* polysaccharide cubosomes have been optimized using response surface methodology.^5^ Optimal preparation conditions were reported in terms of mass percentage ratios between polysaccharide-GMO (1.4%), F127-GMO (9%), and water-GMO (X3) (50%). However, unlike our study, the particle size for the synthesized cubosomes was noted to be 427.7 ± 8.0 nm. The results justified the feasibility and novelty of our research.

### Characterization of Cubosome, Paclitaxel Encapsulation, and Peptide Tagging

As observed from the RSM data, a 95:5 (w/w%) ratio of GMO and DSPE with a 7% F127 w/w ratio gave the optimum size of 110 ± 10 nm and the lowest polydispersity of 0.115 (Figure 2A and Figure S3A). The PDI value in the range of 0.1 to 0.25 indicates that the size distribution is narrow and particles have long-term stability.^23^ A slightly larger size of 129 nm with PDI 0.23 was reported with phytantriol and DPSE-Peg2000 cubosomes.^20^ Paclitaxel (Pxl) is a key drug and a first-line chemotherapeutic treatment for various cancers such as breast cancer, ovarian cancer, nonsmall cell lung cancer, etc.^24^ However, Pxl has some limitations such as low solubility, insufficient tumor penetrating ability, and dose limiting cytotoxicity that make it less effective in clinical applications.^25^ So, we have aimed here to develop a delivery strategy that could overcome these limitations of Pxl. The cubosomes were loaded with 5% w/w Pxl. In our previous work, we studied the efficacy of drug loading in cubosome formulation and found that 5% of the loading gave the highest uptake with a stable particle size. Increasing loading beyond 5%, resulted in the cubosomes losing their stability as a larger size was observed.^4^ It should be noted that Pxl loaded cubosomes have been reported with a size of around 250 nm;^9^ however, our optimized formulations resulted in a much smaller size with lower PDI values. Next the 12-mer peptide (Pep) was tagged on the amine group of DSPE by activating the carboxylic group of the N-terminal amino acid using EDC and NHS. Upon drug encapsulation and peptide tagging, the z-average size of the cubosomes as measured by DLS was observed to be 157 ± 20 nm with a polydispersity of 0.194 ± 0.03 (Figure 2A and Figure S3B). It has been reported that zeta potential values of nanoparticles more than ±30 mV are stable due to electrostatic repulsions.^26^ In our case, the zeta potential value for Cbs-Pxl-Pep was found to be −31.1 mV indicating good stability (Figure S4A). As reported in our previous work, these cubosomes are stable for >21 days at 24 and 37 °C.^3^ The drug release profile of Pxl from Cbs-Pxl-Pep showed a slow and suntained release over 48 h (Figure S4B).

**Figure 2:**
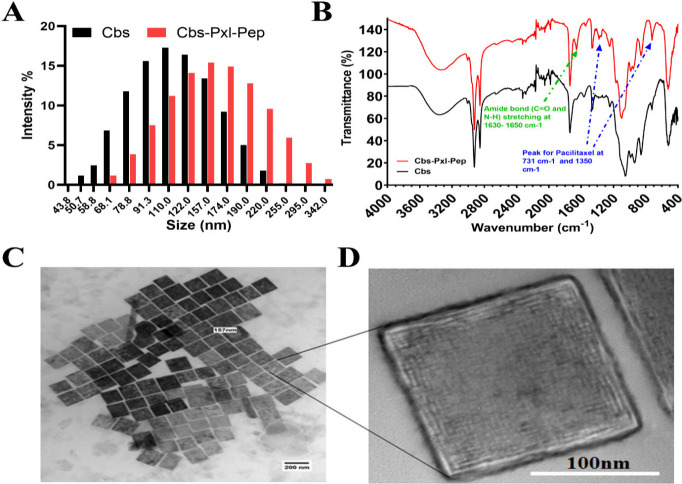
Characterization of cubosomes with drug encapsulation and peptide conjugation. (A) Dynamic light scattering data shows the z-average of bare cubosomes (Cbs) to be 110 ± 10 nm, whereas the z-average of drug encapsulated peptide conjugated cubosomes was 157 ± 20 nm; (B) FTIR spectra confirm the drug encapsulation from the Pxl peak of 731 cm^–1^ and 1350 cm^–1^, whereas peptide conjugation was confirmed from the amide bond peak at 1630–1650 cm^–1^; (C) High resolution Transmission electron microscopy (HR-TEM) image showing the widefield view of Cbs-Pxl-Pep morphology with an average size around 157 nm and (D) magnified view showing the internal mesh structures within the cubosome.

FTIR analysis is a well established method for verifying drug encapsulation and ligand conjugation.^27,28^ We conducted FTIR analysis to validate the peptide tagging and pxl loading (Figure 2B). A peak for the amide bond (C=O and N–H) stretching at 1630–1650 cm^–1^ has been reported in earlier studies.^28^ FTIR data for Cbs-Pxl-Pep shows a sharp presence of a peak around 1630–1650 cm^–1^ confirming the conjugation of the peptide on the cubosomes. There is no significant peak observed in a similar region of 1630–1650 cm^–1^ in the case of bare cubosomes (Cbs) i.e. absence of drug or peptide conjugation. Pxl has significant peaks at 731 cm^–1^ and 1350 cm^–1^ (Figure 2B). These peaks are observed in the Cbs-Pxl-Pep spectra, confirming the uptake of Pxl in the cubosomes. It has been reported that limiting the concentration of drug loading could lead to an increase in the encapsulation efficiency,^28^ so we limited the drug loading to 5%. Paclitaxel can be estimated by a UV–vis spectrophotometer at 231 nm,^29^ and so, the same method was used to study the encapsulation efficiency (EE%). In our previous work, cubosomes had an EE of 82% for a metal organic drug molecule. Similarly, in a recent work, EE of quercetin was observed to be 81.23% in a GMO based cubosome.^30^ In another report, the Pxl EE was found to be 60%. In the case of the present formulation, EE for Pxl was observed to be 80 ± 3% which shows its advantage as a therapeutic carrier.

Finally, the morphology of the Cbs-Pxl-Pep was analyzed using HR-TEM. As observed in our previous work,^3^ the HR-TEM data shows a distinct cubic morphology of the cubosomes around the size of 150 nm (Figure 2C). The internal structure i.e. the mesh like pores, is visible in the magnified view similar to other reports (Figure 2D).^8^

### Selective Uptake of Peptide-Tagged Cubosomes in EGFR +ve Cells

EGFR receptor overexpression is observed in several cancer cells including lung, head and neck, colon, pancreas, breast, ovary, bladder, and kidney and in gliomas.^13^ It has been reported that high expression of EGFR has a poor prognosis in cancer patients.^13^ Cetuximab is a monoclonal antibody specific for the EGFR receptor.^14^ This antibody conjugated delivery of nanotherapeutics to EGFR overexpressing breast cancers has been reported in several recent literature.^14,31^ However, the cost associated with antibody based therapy is beyond the reach of low- and middle income society. As an alternative, we have chosen a 12-amino acid peptide (YHWYGYTPENVI), which has a high binding affinity toward the EGFR receptor.^17^ We aimed to utilize this peptide for the delivery of a chemotherapeutic drug in EGFR overexpressing cancer cells. We selected the breast cancer cell line MDA-MB-468 and noncancerous HEK-293 cells for this study as MDA-MB-468 is one of the cancer cells with very high EGFR expression and HEK-293 is EGFR negative (protein atlas database). However, we reconfirmed EGFR expression of these cells by using immunofluorescence. As observed in Figure 3A using an FITC tagged EGFR binding antibody, MDA-MB-468 cells showed high expression of EGFR, whereas minimal expression was observed in the case of noncancerous HEK-293. The quantitative analysis demonstrated MDA-MB-468 cells overexpress EGFR up to 75% compared to HEK-293 cells (Figure 3B). This is in accordance with the EGFR transcription data from the Protein Atlas database, where MDA-MB-468 cells show approximately 200 times more RNA transcription than HEK-293 cells (Figure S5).

**Figure 3:**
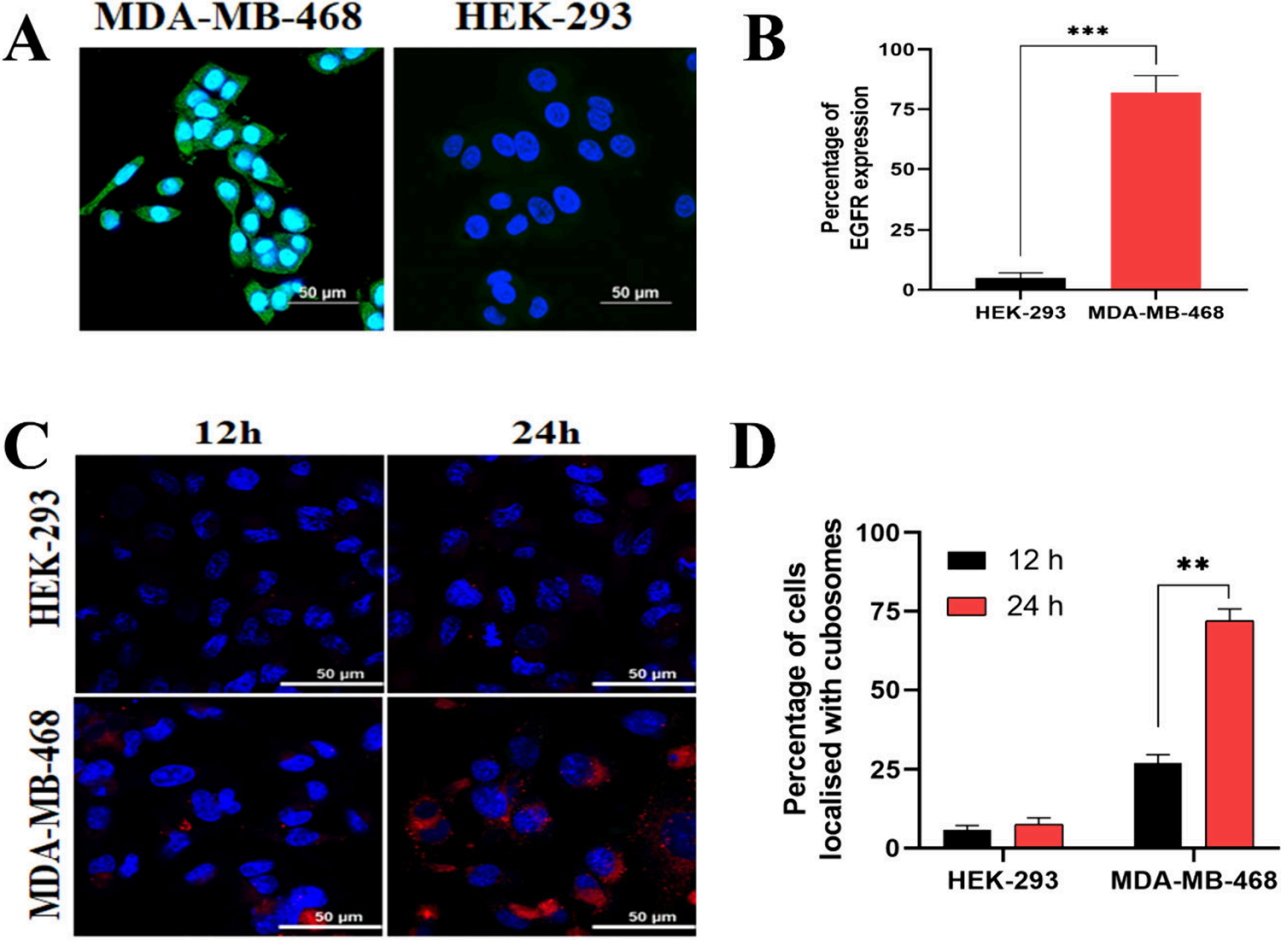
Peptide targeted delivery of cubosome depends on EGFR expression. (A) MDA-MB-468 cells showed 75% increased expression of EGFR (green fluorescence) compared to HEK-293; (B) The quantitative data of EGFR expression; (C) MDA-MB-468 cells showed 25% and 75% uptake of TRITC (red fluorescent dye) encapsulated cubosomes tagged with peptide at 12 and 24 h, respectively, compared to negligible uptake in HEK-293, showing selective delivery of cubosome; (D) Quantitative data of cubosome uptake. The scale bar represents 50 μm. Data represent the mean and standard deviation of three independent experiments.

To study the targeting efficiency of peptide-tagged cubosomes, the cubosomes were incorporated with 0.25% w/w TRITC prior to peptide conjugation. Further, the localization of this red fluorescent cubosomes was studied in the EGFR positive MDA-MB-468 and EGFR negative noncancerous HEK-293 cells at 12 and 24 h, respectively. The EGFR-positive cells (MDA-MB-468) showed specific uptake of red fluorescent cubosomes in the cytoplasmic region, as observed by confocal microscopy (Figure 3C), demonstrating EGFR dependent uptake of the peptide-tagged cubosomes. At 12 h, uptake was observed in around 25% of the MDA-MB-468 cells, which increased to 75% at 24 h (Figure 3D). On the contrary, there was negligible uptake of cubosomes in EGFR negative HEK-293 cells at 12 h. Additionally, there was no significant increase in the uptake of cubosomes with increasing time (24 h) for HEK-293 cells. These data suggest that the 12mer peptide selectively delivers the payload of pxl encapsulated cubosomes in EGFR overexpressing cancer cells.

### Peptide-Tagged Cubosomes Selectively Eliminate EGFR Expressing Cancer Cells

Although reports suggest that GMO-based cubosomes are biocompatible, due to the unique formulation of cubosomes in our work, we evaluated the toxicity of bare Cbs using the MTT assay. It was observed that bare Cbs did not show any significant cytotoxicity in either of the cell lines at a concentration up to 100 μg/mL (Figure S7). Next, we evaluated the efficacy of the targeted delivery of paclitaxel in peptide-tagged cubosomes using the MTT assay. Both EGFR positive MDA-MB-468 and EGFR negative HEK-293 cell lines were treated with varying concentrations (0–100 μg/mL) of drug-loaded untargeted cubosomes (Cbs-Pxl) or drug-loaded peptide-tagged cubosomes (Cbs-Pxl-Pep), and cell viability was assessed at 12 and 24 h. Untargeted drug loaded cubosomes (Cbs-Pxl) showed slight cytotoxicity at the highest concentrations (for example at 12 h around 90% viability and at 24 h 84% viability were observed at 100 μg/mL), indicating nonspecific uptake of the Cbs-Pxl in both cell lines (Figure S8), whereas Cbs-Pxl-Pep showed a significant reduction in cell viability in the EGFR-expressing cells MDA-MB-468 (Figure 4A-B). On the contrary, relatively minimal toxicity was observed in the case of EGFR negative HEK-293 cells, which was similar to the toxicity caused by Cbs-Pxl. For example, at 12 h (Figure 4A), 60 μg/mL Cbs-Pxl-Pep showed viability of 71% and 87% in MDA-MB-468 cells and HEK-293 cells, respectively (p = 0.0005), whereas at 24 h (Figure 4B), 60 μg/mL Cbs-Pxl-Pep showed viability of 47% and 83% in MDA-MB-468 cells and HEK-293 cells respectively (*p* < 0.0001). These findings correlate with the *in vitro* localization data (Figure 3C), as EGFR-expressing cells showed selective uptake of the peptide-tagged cubosomes, thereby causing high levels of paclitaxel toxicity. The IC_50_ value of Cbs-Pxl and Cbs-Pxl-Pep was analyzed for 24 h treatment using a nonlinear regression curve (Figure 4C–D). As seen in Figure 4C, the IC_50_ for Cbs-Pxl-Pep was found to be 53.72 μg/mL and 119.6 μg/mL for MDA-MB-468 cells and HEK-293 cells respectively (*p* < 0.0001), which indicates the successful targeting efficiency of the 12mer peptide to selectively deliver the Cbs-Pxl in EGFR expressing cancer cells. On the contrary, the IC_50_ value for Cbs-Pxl was significantly higher in both cells indicating minimal nonspecific uptake in the absence of peptide (Figure 4D). Similar targeting efficiency of cubosomes has been reported in our recent work and by another group using hyaluronic acid to target CD44 receptor overexpressing cancer cells.^4,32^ Further, the IC_50_ dose of Cbs-Pxl-Pep in MDA-MB-468 i.e. 53 μg/mL was treated in both cells, and the morphology of cells/nucleus was observed with Hoechst 33342 staining of the nucleus (Figure 4E). After 12 h of treatment, there was a change of cell morphology in 25% of MDA-MB-468 cells, which further increased to 90% of cells after 24 h of treatment, which is a probable indication of apoptosis induced by paclitaxel. On the contrary, HEK-293 cells showed a negligible effect of Cbs-Pxl-Pep as seen from the cell morphology. Paclitaxel alone shows no specificity against cancer cells as it is also cytotoxic for noncancerous HEK-293 cells at low doses (Figure S9). To further validate the specificity of the Cbs-Pxl-Pep toward the EGFR receptor, two more cell lines with different levels of EGFR expression based on RNA transcription were evaluated. As noted from the Protein Atlas database, MDA-MB-231 cells have a moderate level of EGFR RNA transcription, whereas MCF-7 cells are EGFR negative (Figure S5). MCF-7 and MDA-MB-231 cells were treated with Cbs-Pxl-Pep for 12 h and 24 h, and the IC50 value was noted using the MTT assay. Cbs-Pxl-Pep showed a similar cell cytotoxicity effect in MDA-MB-231 as MDA-MB-468 cells with an IC_50_ value of 85.8 μg/mL. The slight increase in the IC_50_ value compared to that in MDA-MB-468 cells could be attributed to the reduced expression of EGFR in MDA-MB-231 compared to MDA-MB-468. On the contrary, MCF-7 cells similar to HEK-293 had much higher survivability toward Cbs-Pxl-Pep with an IC_50_ value of 137.2 μg/mL. For further studies, we chose the EGFR +ve cancerous MDA-MB-468 and EGFR negative noncancerous HEK-293 as our cell line models.

**Figure 4:**
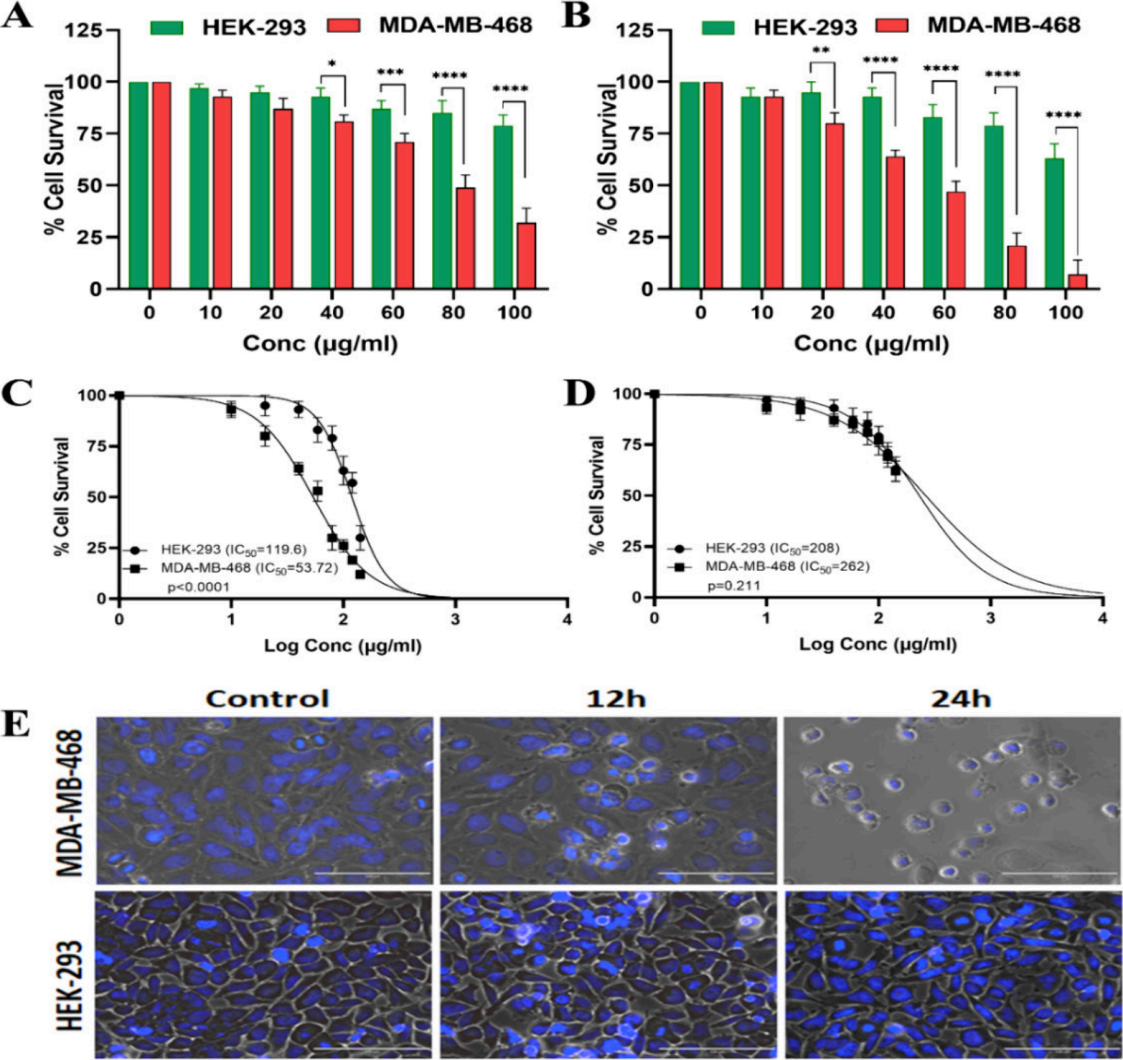
Drug encapsulated, Peptide-tagged cubosomes selectively kill EGFR expressing cancer cells. (A) Cells were treated with various doses of Cbs-Pxl-Pep for 12 h. HEK-293 cells showed a negligible effect even at the highest concentration; however, MDA-MB-468 cells showed significantly reduced survivability (****p < 0.0001, ***p < 0.001, *p < 0.05 using two-way ANOVA) with increasing concentration; (B) The response was more significant (****p < 0.0001, **p < 0.01 using two-way ANOVA) at 24 h of treatment where MDA-MB-468 cells showed more than 50% of cell death compared to HEK-293 having 90% survival when treated with 60 μg/mL Cbs-Pxl-Pep; (C) A nonlinear regression curve was used to determine the IC_50_ for 24 h of treatment, showing a significant difference between the two cell lines. The IC_50_ of MDA-MB-468 was 53 μg/mL; (D) Cbs-Pxl showed no significant difference between IC_50_ values of the two cell lines indicating negligible nonspecific uptake in the absence of peptide conjugation; (E) Cells after treating with IC_50_ of Cbs-Pxl-Pep show changes in cell morphology in MDA-MB-468, indicating apoptosis induction, but no change in HEK-293 was observed. The scale bar represents 100 μm. Data represents the mean and standard deviation of three independent experiments.

### Peptide-Tagged Drug Loaded Cubosomes Show Mitochondrial Damage Induced Apoptosis in the Targeted Cells

Nano formulation of paclitaxel has been reported previously to induce mitochondrial membrane depolarization leading to apoptosis in several cancers.^33^ Jc-1 is commonly used to analyze the mitochondrial potential as it is a dual fluorescent dye that accumulates in healthy mitochondria in its aggregate form, emitting red fluorescence, while in depolarized mitochondria, this dye transforms into its monomeric form, giving green fluorescence. In this study, when cells were treated with the IC_50_ dose (i.e., 53 μg/mL) of Cbs-Pxl-Pep, 65% of MDA-MB-468 cells had depolarized mitochondria at 12 h which increased to 92% at 24 h (*p* < 0.01) (Figure 5A). On the contrary, HEK-293 cells had no significant effect on mitochondria either at 12 or 24 h post treatment with Cbs-Pxl-Pep (Figure 5A). Drug induced mitochondrial depolarization is reported to cause apoptosis mediated cell death.^33^ Next, we analyzed the cells for apoptosis using the FACS method.^34^ MDA-MB-468 cells treated with an IC_50_ dose of Cbs-Pxl-Pep showed a significant increase in apoptosis with increasing time points. At 12 h, cells had a 13% apoptotic population (cumulative of early and late apoptotic cells) which increased to 49% at 24 h (<0.0001), whereas HEK-293 cells had no significant increase in apoptosis, with only 6% cells in the apoptotic phase at 24 h (Figure 5B). Apoptosis was also analyzed for untargeted delivery with Cbs-Pxl (Figure S13), where no significant increase in apoptosis population was observed in either cell line, which was in correlation with the MTT data (Figure 4A-B).

**Figure 5:**
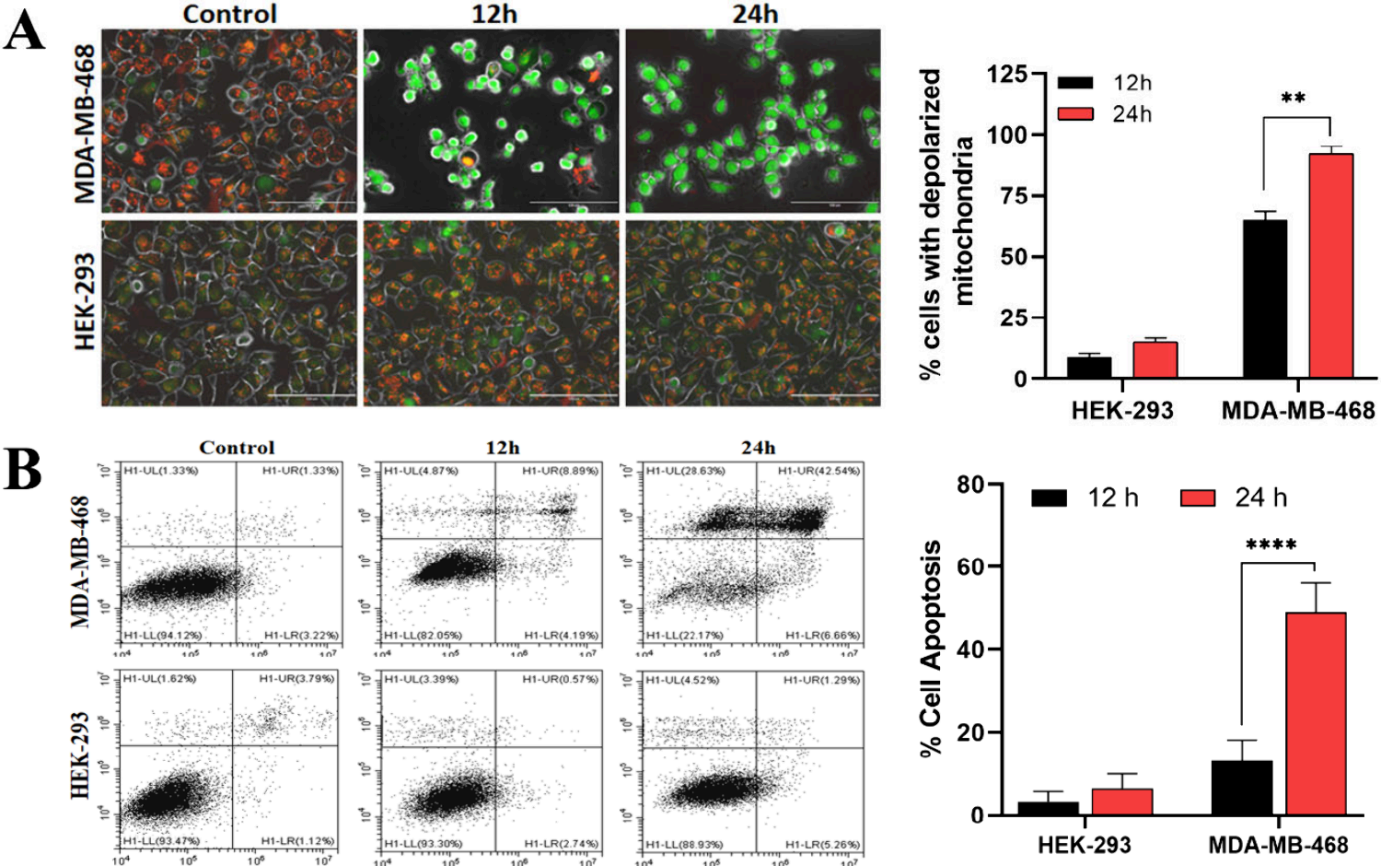
Cbs-Pxl-Pep causes mitochondrial membrane depolarization and apoptosis in EGFR expressing cancer cells. (A) 53 μg/mL Cbs-Pxl-Pep treatment showed induction of mitochondrial membrane depolarization in MDA-MB-468 (as indicated by shift of fluorescence from red to green), but no significant change in HEK-293 was observed. The change in depolarized mitochondria significantly increased from 65% to 92% in MDA-MB-468 cells (**p < 0.01, using two-way ANOVA) at 12 and 24 h respectively, but no change was observed in HEK-293. Scale bar represents 100 μm; (B) Flow cytometric analysis showed a significant increase in apoptotic cell populations in IC_50_ treated MDA-MB-468 cells at 24 h (****p < 0.0001, using two-way ANOVA) compared to a negligible change in the case of HEK-293. Data represents the mean and standard deviation of three independent experiments.

### Peptide-Tagged Drug Loaded Cubosomes Cause Specific Cytotoxicity in 3D Spheroids of EGFR Expressing Cells

3D spheroids offer a more accurate representation of *in vivo* tumors compared to traditional 2D monolayer cultures.^3^ They mimic the complex tumor architecture, cell–cell interactions, and nutrient gradients of actual tissues, enhancing the relevance of drug response and cancer biology studies. Spheroids demonstrate better simulation of oxygen, nutrient, and waste diffusion, leading to more physiologically relevant cellular behaviors and drug resistance patterns.^3,32^ Additionally, they allow for the study of hypoxic conditions and the extracellular matrix’s role in cell signaling and growth, providing a more comprehensive model for therapeutic testing and understanding tumor biology.^32^ It is commonly observed that spheroids due to their complex extracellular matrix are more resistant to drugs and thus serve as a better model to understand how a drug’s efficacy would vary in an actual solid tumor.^3,4^ We further evaluated the efficacy of Cbs-Pxl-Pep in 3D spheroids of HEK-293 and MDA-MB-468. Spheroids were treated with IC_50_ (53 μg/mL) of Cbs-Pxl-Pep for 24 and 48 h. Propidium iodide was used to analyze spheroid viability, along with nuclear staining dye Hoechst 33342. Here, Propidium iodide stain indicates dead cells as this dye cannot permeabilize viable cells, whereas Hoechst 33342 could permeate both viable and nonviable cells, indicating all cells present in the spheroid.^3,4^ Post treatment with Cbs-Pxl-Pep, it was observed that viability was 65% after 24 h and 13% (p = 0.0001) at 48 h, in the case of the MDA-MB-468 spheroid.

In the case of HEK-293 cells, only minor and nonsignificant reductions in spheroid viability were observed at either time point (Figure 6A). This suggests that Cbs-Pxl-Pep had effective access to the spheroid’s interior. In our monolayer culture study, Cbs-Pxl-Pep showed apoptosis mediated cell death; therefore, we analyzed the spheroids for apoptosis with and without treatment with Cbs-Pxl-Pep for 48 h. Caspase 3 is an apoptotic protein marker, and in the event of apoptosis, there is a decrease in full-length caspase 3 expression.^4^ From the Western blot analysis of HEK-293 spheroids (Figure 6B), there was no significant reduction of caspase 3 after treatment with Cbs-Pxl-Pep for 48 h. On the contrary, in the case of MDA-MB-468 cells, there was around 50% reduction of caspase 3 (P = 0.0154), indicating the induction of apoptosis post Cbs-Pxl-Pep treatment (Figure 6B). Thus, it was evident that the targeted delivery of paclitaxel loaded cubosomes by the 12mer peptide successfully induced selective apoptosis, potentially eliminating EGFR-expressing cancer cells without causing any toxicity to noncancerous or EGFR negative cells *in vitro*.

**Figure 6:**
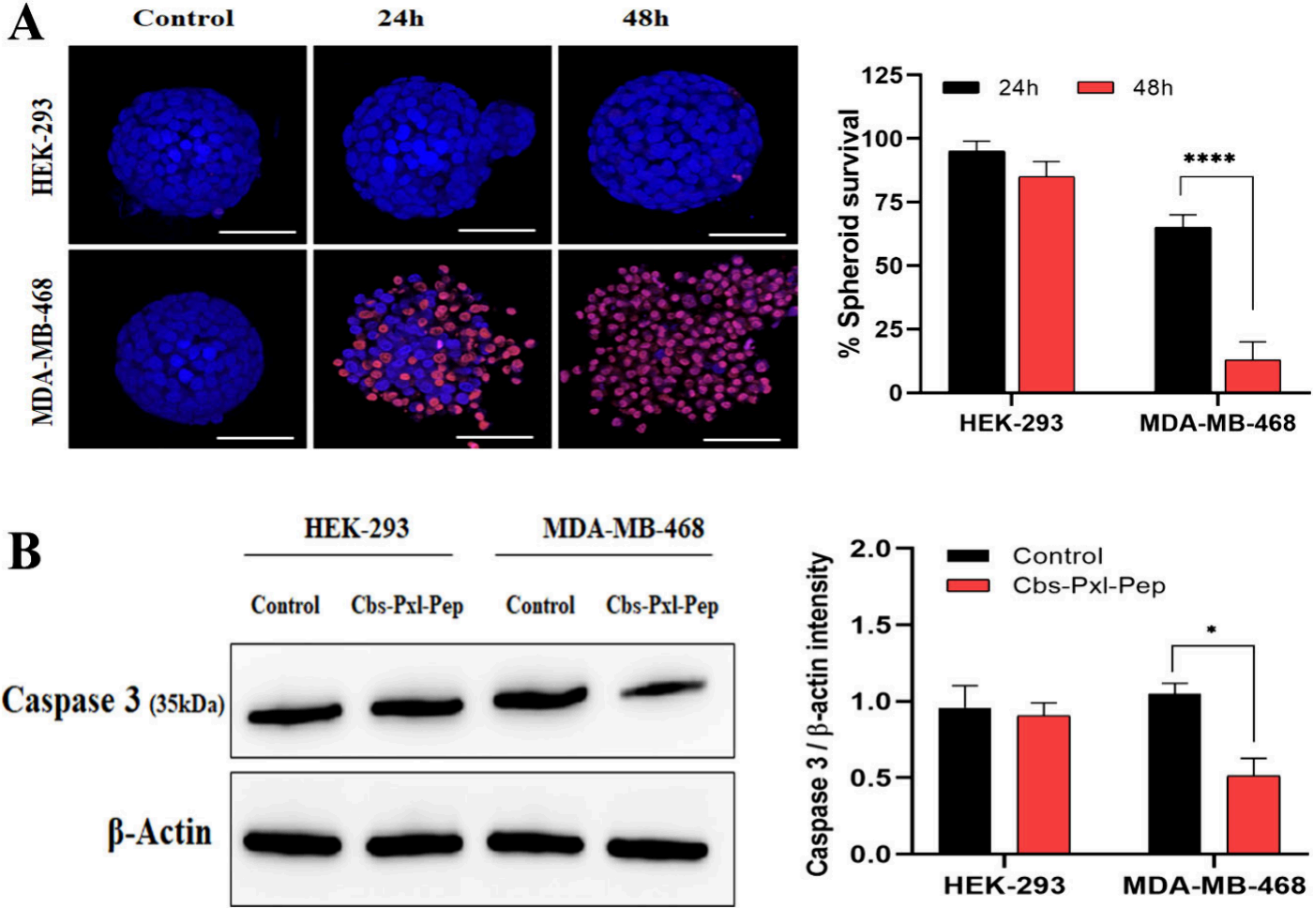
Cbs-Pxl-Pep causes apoptosis mediated cell death specifically in EGFR expressing spheroids. (A) 53 μg/mL Cbs-Pxl-Pep showed a significant increase in cell death between 12 and 24 h in the MDA-MB-468 spheroids (****p < 0.0001, using two-way ANOVA) as indicated by red fluorescence of propidium iodide from dead cells (blue fluorescence is from nuclear staining with Hoechst 33342), whereas no sign of cell death was observed in the case of HEK-293 spheroids. Scale bar represents 200 μm; (B) From Western blot analysis, after treatment with Cbs-Pxl-Pep, reduction of caspase 3 was observed in the case of MDA-MB-468 (*p < 0.05, using two-way ANOVA) indicating induction of apoptosis in EGFR +ve spheroids. However, no apoptosis or cytotoxicity was noted in HEK-293 spheroids showing the absence of nonspecific cytotoxicity of Cbs-Pxl-Pep. Data represent the mean and standard errors of three independent experiments.

### EGFR Targeted Delivery of the Drug Shown to Restrict Tumor Growth Alongside Reduced Cytotoxicity *in Vivo*

Our next aim was to analyze the therapeutic efficacy of Cbs-Pxl and Cbs-Pxl-Pep in a tumor model *in vivo*. Before the cubosome formulation was administered *in vivo*, it was essential to check for toxicity using a hemolysis assay. A hemolysis assay is a standard procedure to check for the safety of a drug molecule before administering it *in vivo*.^35^ Different concentrations of Cbs-Pxl and Cbs-Pxl-Pep (50, 100 μg/mL) were treated with RBC isolated from balb/c mice (Figure 7A). 1% Triton X was used as a positive control. It was observed that at the highest concentration i.e. 100 μg/mL Cbs-Pxl, 7% hemolysis was observed. The same concentration of Cbs-Pxl-Pep showed 3.5% hemolysis (Figure 7A). This shows the biocompatibility of the cubosome formulation. Next, the cubosome formulations were administered in tumor bearing mice. Mice breast cancer cell line 4T1 was used to develop tumors in balb/c female mice, and then the mice were divided randomly into three groups with 6 mice in each group. Each group received intravenous administration of saline (untreated control), Cbs-Pxl (nontargeted), or Cbs-Pxl-Pep (targeted) at 1, 3, and 5 days, respectively. The tumor volume was monitored throughout the duration of the experiment. The tumor volume continued to grow at a steady rate in the control and nontargeted group (Figure 7C). The mean tumor volume on the 12th day was observed to be 410 mm^3^, 370 mm^3^, and 200 mm^3^ in the control, nontargeted, and targeted groups respectively (Figure 7C). As observed, there was restricted tumor growth in the targeted group compared with the rest. At the end of the 24th day, the tumor volume was 770 mm^3^ and 675 mm^3^ in the control and nontargeted group respectively (Figure 7D-F). Whereas in the targeted group, a significant reduction in tumor growth of 330 mm^3^ was observed (Figure 7E). With respect to day 1, on day 24 the fold increase in mean tumor volume was 8.6 times the control group (Figure 7G), and 6.9 times the nontargeted group. Whereas, in the targeted group, the fold increase was significantly reduced 3.5 times (Figure 7G). This clearly indicates that upon targeted delivery of drug (i.e., Pxl) loaded cubosomes, uptake of Cbs-Pxl-Pep to the tumor microenvironment was enhanced, which resulted in significantly enhanced inhibition of tumor growth. One step further, tumor weights were also measured on day 24 (Figure 7H), and it was observed that the targeted group had the least weight of tumor, i.e., 0.8 g, compared to the control and nontargeted group, which had significantly increased weight of tumor, i.e., 2.2 and 1.9 g, respectively (Figure 7H).

**Figure 7:**
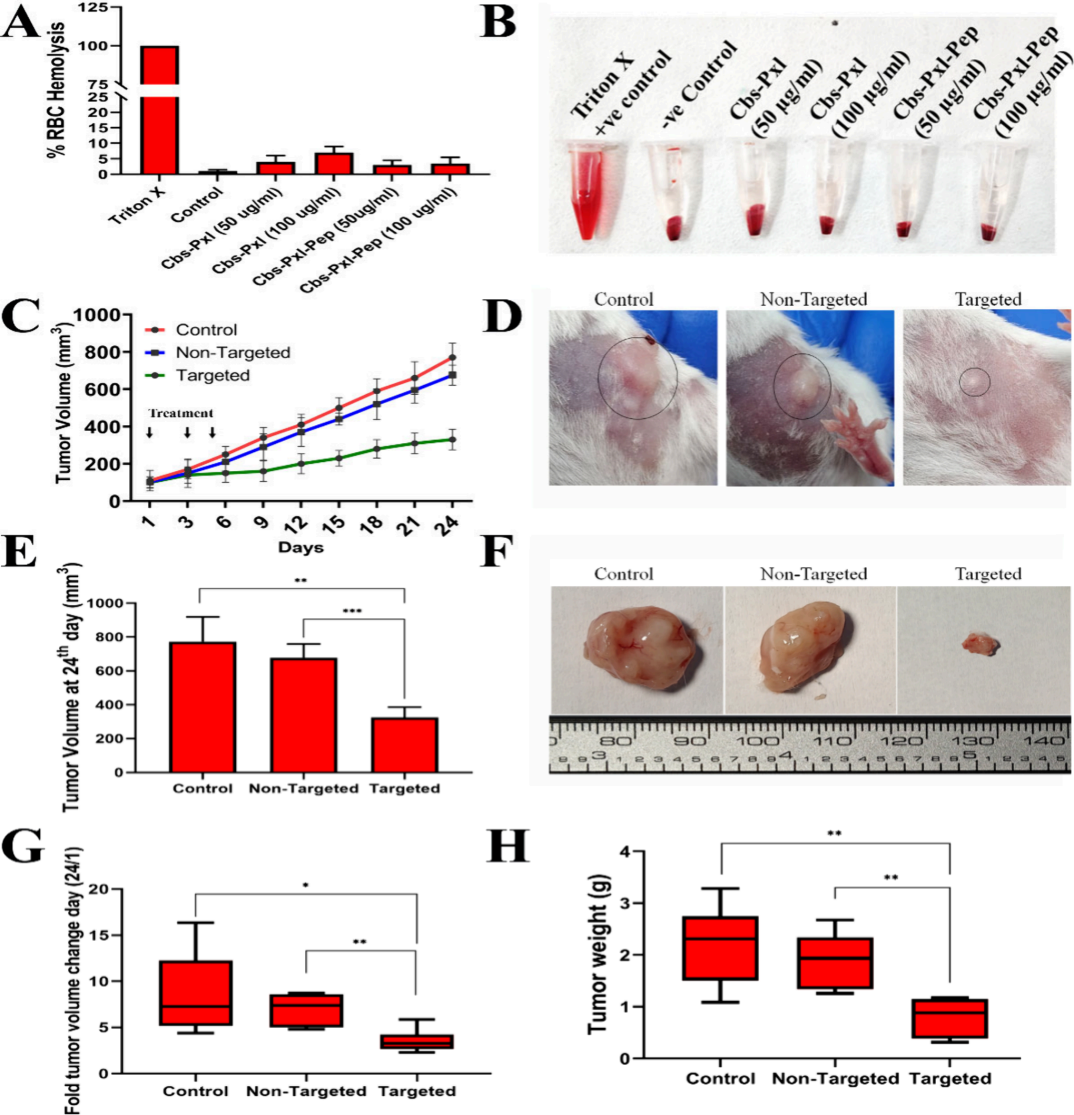
Targeted delivery of drug showed enhanced efficacy in reducing tumor growth. (A-B) Cbs-Pxl-Pep and Cbs-Pep did not show any significant signs of hemolysis ex-vivo up to 100 μg/mL concentration; (C) The change in tumor volume during the course of experiment in all three groups showing Cbs-Pxl-Pep having the least tumor growth compared to Cbs-Pxl and the control group; (D-F) Tumor volume as observed at day 24 in the three groups. Cbs-Pxl-Pep treatment had a mean tumor volume of 325 mm^3^ which was significantly smaller than Cbs-Pxl which had 678 mm^3^ and the control group having 772 mm^3^ (**p < 0.01, ***p < 0.001 using unpaired *t* test). (G) Mean of tumor volume fold change (at day 24 compared to day 1) was found to be the least in the case of the Cbs-Pxl-Pep treated group which was 3.5 compared with Cbs-Pxl which was 6.9 and 8.6 times the untreated control group (*p < 0.05, **p < 0.01 using unpaired *t* test). (H) Cbs-Pxl-Pep treated group had the least tumor weight of 0.8 g compared to the Cbs-Pxl (untargeted) group and untreated control group having 1.9 and 2.2 g respectively (**p < 0.01 using unpaired *t* test).

It is essential to check for toxicities of drug formulations in the mice, which could occur due to the nano formulation administration. It was observed that the mice in the targeted group had improved survivability compared to the control and nontargeted group (Figure 8A). In the control group (saline), four mortalities were observed on the 12th, 18th, 21st, and 24th days, respectively. In the nontargeted group (Cbs-Pxl), three mortalities were observed on the 12th, 14th, and 18th days, respectively. Whereas in the targeted group (Cbs-Pxl-Pep), one mortality was observed on the 18th day (Figure 8A). Thus, peptide mediated targeted delivery of the Pxl improved the overall health of the mice. Another parameter for drug toxicity is a change in body weight. The body weight is measured, and a change of 25% in body weight is considered an adverse effect.^3^ We observed that in the case of the targeted or nontargeted group there was no significant change in body weight indicating the absence of any adverse effect caused by the administration of Cbs-Pxl or Cbs-Pxl-Pep (Figure 8B). Further, we also analyzed kidney and liver tissue using H&E staining and found there was no sign of abnormality or toxicity in the Cbs-Pxl-Pep treated i.e. targeted group (Figure 8C–D). Tumor tissues of the three groups were also analyzed to confirm tumor cell death as a positive indicator for targeted delivery of Pxl (Figure 8E). In the case of Cbs-Pxl-Pep, there was a clear sign of cell death in the tumor tissue, confirming the successful delivery of the drug to the tumor microenvironment upon peptide targeting (Figure 8E). This indicates that the peptide-tagged cubosomes can effectively deliver the drug to the tumor site to inhibit tumor growth without causing toxicity in the body.

**Figure 8:**
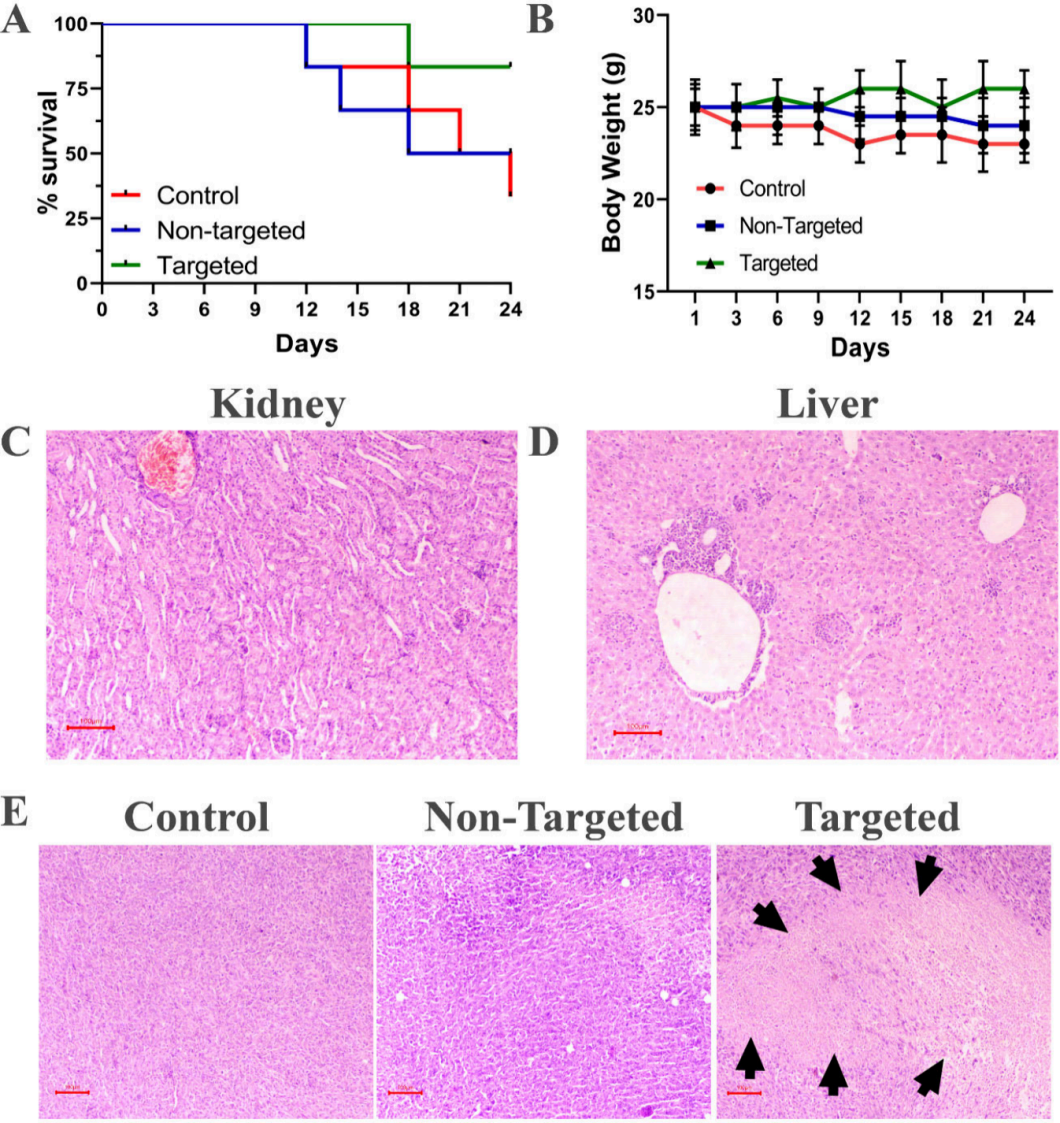
Targeted delivery of paclitaxel using the cubosome formulation did not show any signs of toxicity but had enhanced tumor tissue cell death. (A) Cbs-Pxl-Pep treated group had 83% survivability of mice compared to 50% and 33% survivability in Cbs-Pxl treated (untargeted) and in untreated control respectively; (B) No significant change in body weight was observed in the Cbs-Pxl-Pep or Cbs-Pxl treated group; (C–D) No signs of tissue damage or toxicity were observed from the H&E staining of Kidney and Liver from the Cbs-Pxl-Pep treated group; (E) Histology tissue section analysis of tumor tissue showed increased cell death in the Cbs-Pxl-Pep treated group (indicated in black heads) compared to Cbs-Pxl and the control group. Scale bar represents 100 μm.

## Conclusions

This study establishes a robust and rational framework for the optimization of cubosome-based nanocarriers using response surface methodology (RSM), demonstrating how formulation and process parameters can be systematically tuned to achieve nanoscale architectures that are ideally suited for targeted drug delivery. The strong statistical significance of the model and identification of critical parameter interactions underscore the reliability and translational relevance of this approach. Importantly, the optimized cubosomes fall within a size regime widely recognized to favor enhanced tumor accumulation, reinforcing the practical applicability of the developed formulation strategy.

A major strength of this work lies in the successful integration of active targeting of cubosome-based drug delivery, an area that has remained relatively underdeveloped, despite the intrinsic advantages of lipidic nanocarriers. To the best of our knowledge, this is the first comprehensive demonstration of peptide-tagged cubosomes designed to selectively target EGFR-expressing cancer cells. Notably, the targeted cubosomes significantly enhanced therapeutic efficacy while simultaneously minimizing the cytotoxic effects of potent chemotherapeutic agents, such as paclitaxel, addressing one of the most critical limitations of current cancer chemotherapy. Furthermore, the significant inhibition of tumor growth observed *in vivo*, in the absence of systemic toxicity, strongly supports the translational promise of this strategy. Compared with existing antibody-based therapies targeting EGFR, such as Cetuximab, the peptide-functionalized cubosome platform offers a potentially cost-effective, scalable, and versatile alternative.

Although only a limited number of studies have explored cubosomes for chemotherapeutic delivery and even fewer have incorporated active targeting strategies, the present work significantly expands the biomedical scope of cubosome nanocarriers. By combining statistical formulation optimization with biologically validated targeting and therapeutic efficacy, this study positions cubosome-based nanomedicine as a powerful and adaptable platform for the treatment of EGFR-expressing cancers and potentially other malignancies. These findings represent an important contribution to the field of targeted nanotherapeutics and lay a strong foundation for future translational and clinical investigations.

## Supplementary Material



## Data Availability

The data used in this research article will be made available upon request.

## Notes

ANOVA table showing respective sum of squares, degree of freedom, mean square, F-value, and p-value for response of parameters on cubosome size; Contour plots and 3D mesh diagram showing interaction effects on cubosome particle size; DLS data showing hydrodynamic diameter of cubosome; Zeta potential data showing particle charge; Drug release profile; EGFR expression on various cell lines; Cubosome uptake study as observed from TRITC fluorescence; cell cytotoxicity study of bare cubosome, drug encapsulated cubosome, and peptide tagged drug encapsulated cubosome on MDA-MB-468 and HEK-293 cells; Mitochondrial membrane potential change and nucleus morphology change study upon treatment of drug encapsulated cubosome; Apoptosis measurement of cells as observed from flow cytometry (PDF)
