# Supplementary material for: Design and Synthesis of Peptide-Tagged Cubosome Nanocarriers for the Targeted Delivery of Paclitaxel in EGFR Overexpressing Breast Cancer
Source: ACS Biomater Sci Eng. 2026 Feb 19;12(3):1633–46. doi: 10.1021/acsbiomaterials.5c02193 (PMC12976991; doi:10.1021/acsbiomaterials.5c02193)
Supplement: Supplementary file 1 [file ab5c02193_si_001.pdf]

SUPPORTING INFORMATION FOR PUBLICATION

Design and synthesis of peptide-tagged cubosomes nanocarriers for the targeted delivery of paclitaxel in EGFR over-expressing breast cancer

Arindam Pramanik[1,2],*, Riya Rani[2], Bhavna Jha[1], Devlina Das Pramanik[1], Prashant Mishra[2,*]

[1]Amity Institute of Biotechnology, Amity University, Noida 201301, India

[2]Department of Biochemical Engineering and Biotechnology, Indian Institute of Technology Delhi, New Delhi, 110016, India

***Corresponding authors:**

Dr. Arindam Pramanik, Amity Institute of Biotechnology, Amity University, Noida 201301, India.

Email: arindampramanik87@gmail.com

Prof. Prashant Mishra, Department of Biochemical Engineering and Biotechnology, Indian Institute of Technology Delhi, Hauz Khas, New Delhi 110016, India.

Email: pmishra@dbeb.iitd.ac.in

Table S1: ANOVA table showing respective sum of squares, degree of freedom, mean square, F-value and p-value for response of parameters on as-synthesized cubosome size

Source	*Sum of Squares*	*df*	*Mean Square*	*F-value*	*p-value*	
Model	26620.48	14	1901.46	24.51	< 0.0001	significant
A-GMO Dosage	14.08	1	14.08	0.1815	0.6766	
B-DSPE Dosage	78.37	1	78.37	1.01	0.3319	
C-F127 Dosage	611.56	1	611.56	7.88	0.0140	
D-Sonication Time	560.33	1	560.33	7.22	0.0177	
AB	28.44	1	28.44	0.3666	0.5546	
AC	21.78	1	21.78	0.2807	0.6046	
AD	78.03	1	78.03	1.01	0.3330	
BC	10.03	1	10.03	0.1292	0.7246	
BD	1.36	1	1.36	0.0175	0.8965	
CD	49.00	1	49.00	0.6315	0.4401	
A^2	15538.76	1	15538.76	200.27	< 0.0001	
B^2	4049.55	1	4049.55	52.19	< 0.0001	
C^2	2515.92	1	2515.92	32.43	< 0.0001	
D^2	13469.67	1	13469.67	173.60	< 0.0001	

R^2: 0.9608; Adequate Precision: 16.5240; Recommended Transformation: None; Significant Model Terms: Single Factors- C (F127 Dosage), D (Sonication Time); Quadratic Factors: A^2, B^2, C^2, D^2

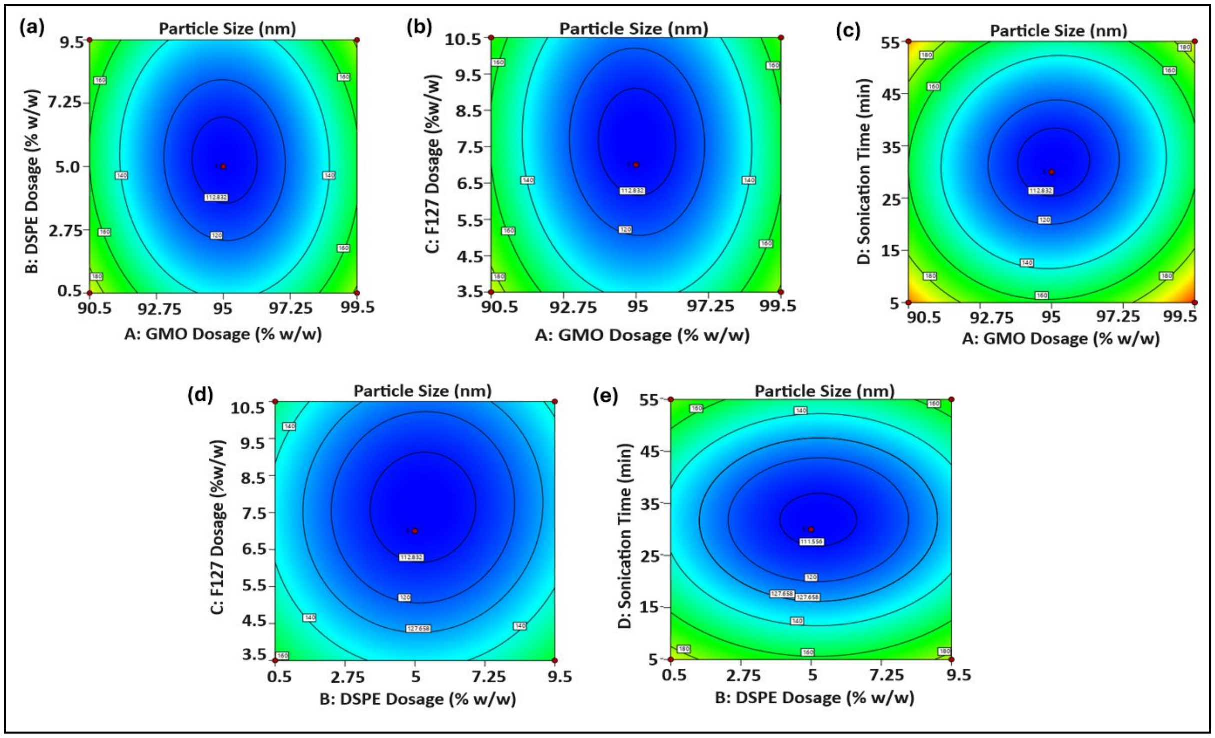

Figure S1: Contour plots showing the interaction effects of single factors on synthesized cubosome particle sizes.

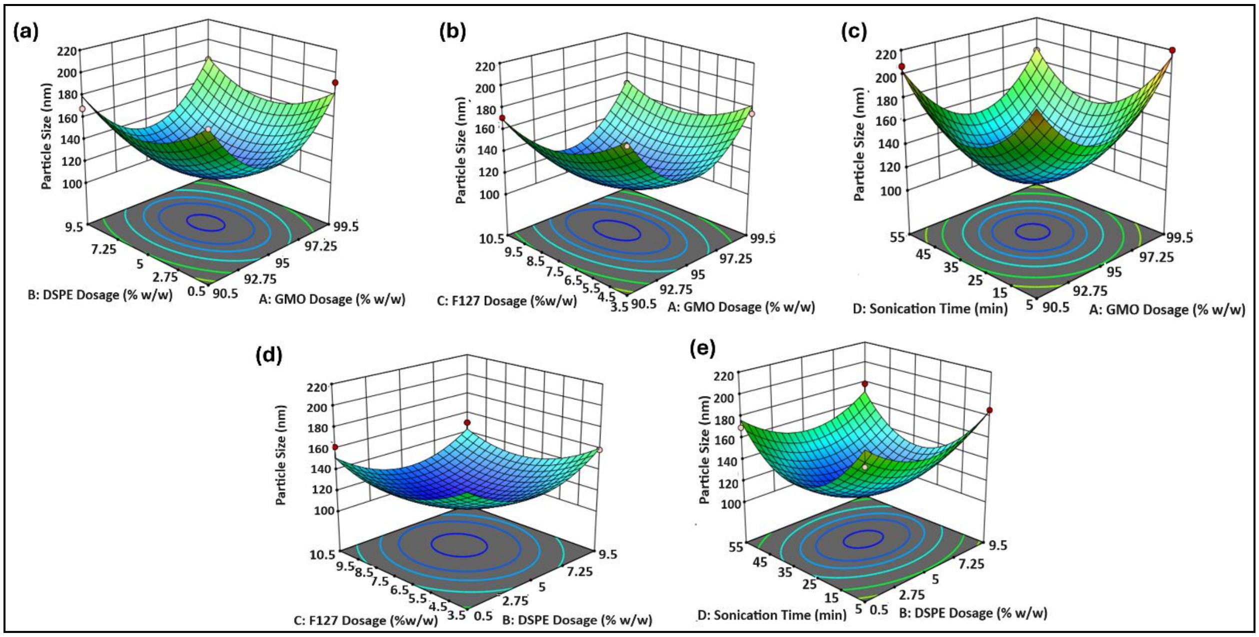

Figure S2: 3D mesh diagram plots showing the interaction effects of single factors on synthesized cubosome particle sizes a to e.

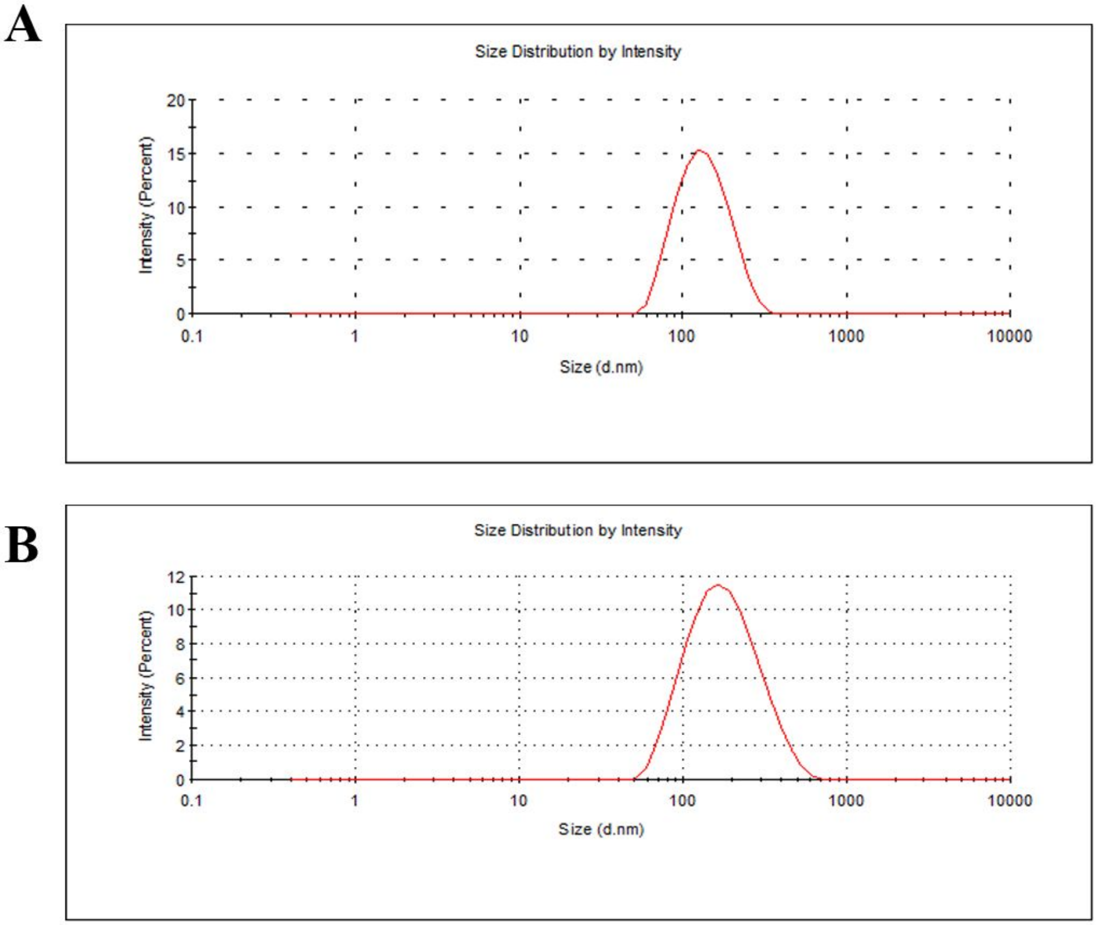

Figure S3: Dynamic light scattering data showing the hydrodynamic diameter (z-average) of (A) bare cubosomes (110 nm) and (B) cubosomes encapsulated with paclitaxel and conjugated with peptide (257 nm).

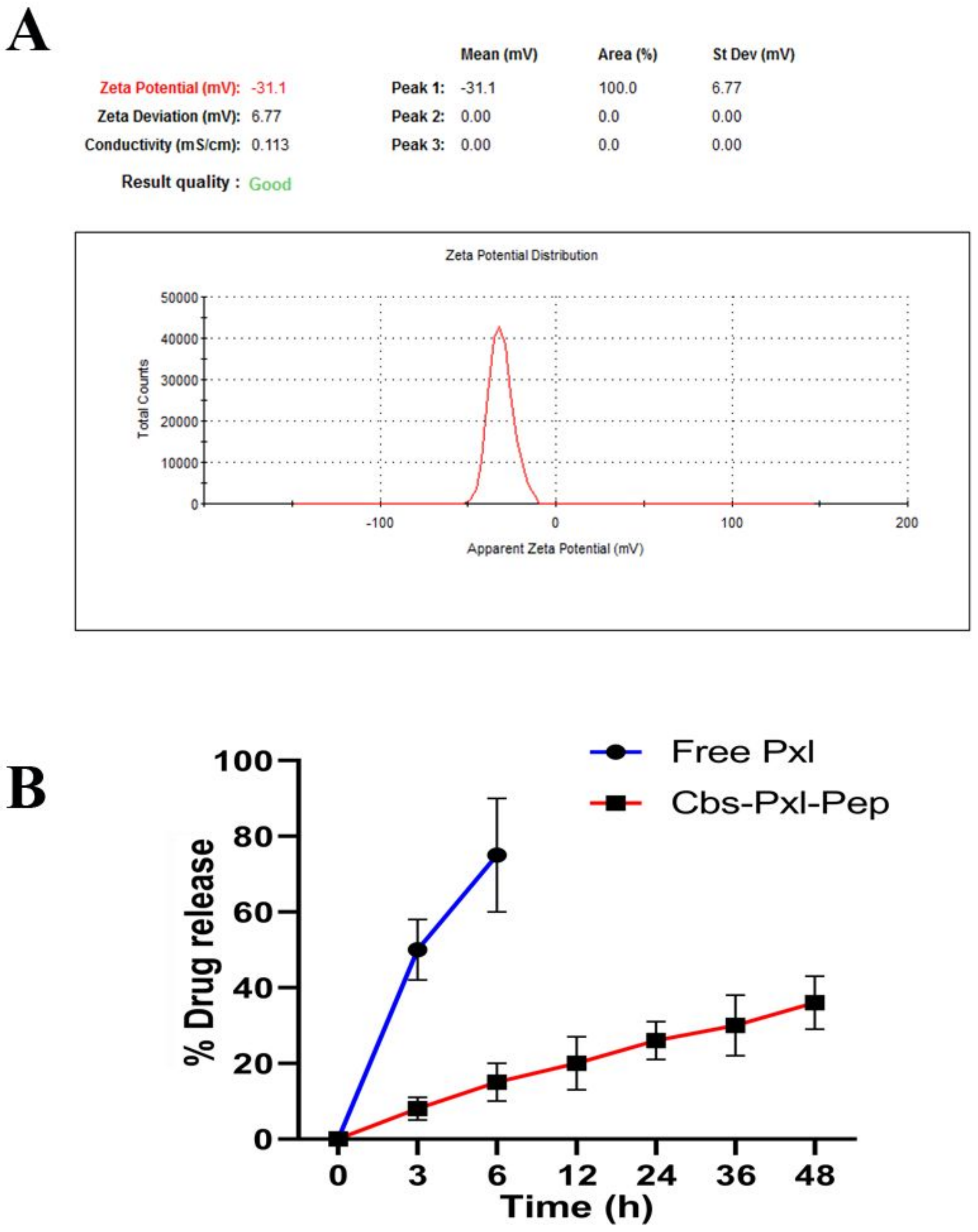

Figure S4: (A) Zeta potential measurement of Cbs-Pxl-pep was found to be -31.1mV. (B) Drug release profile showing burst release of free paclitaxel whereas slow and sustained release of Pxl from cubosome.

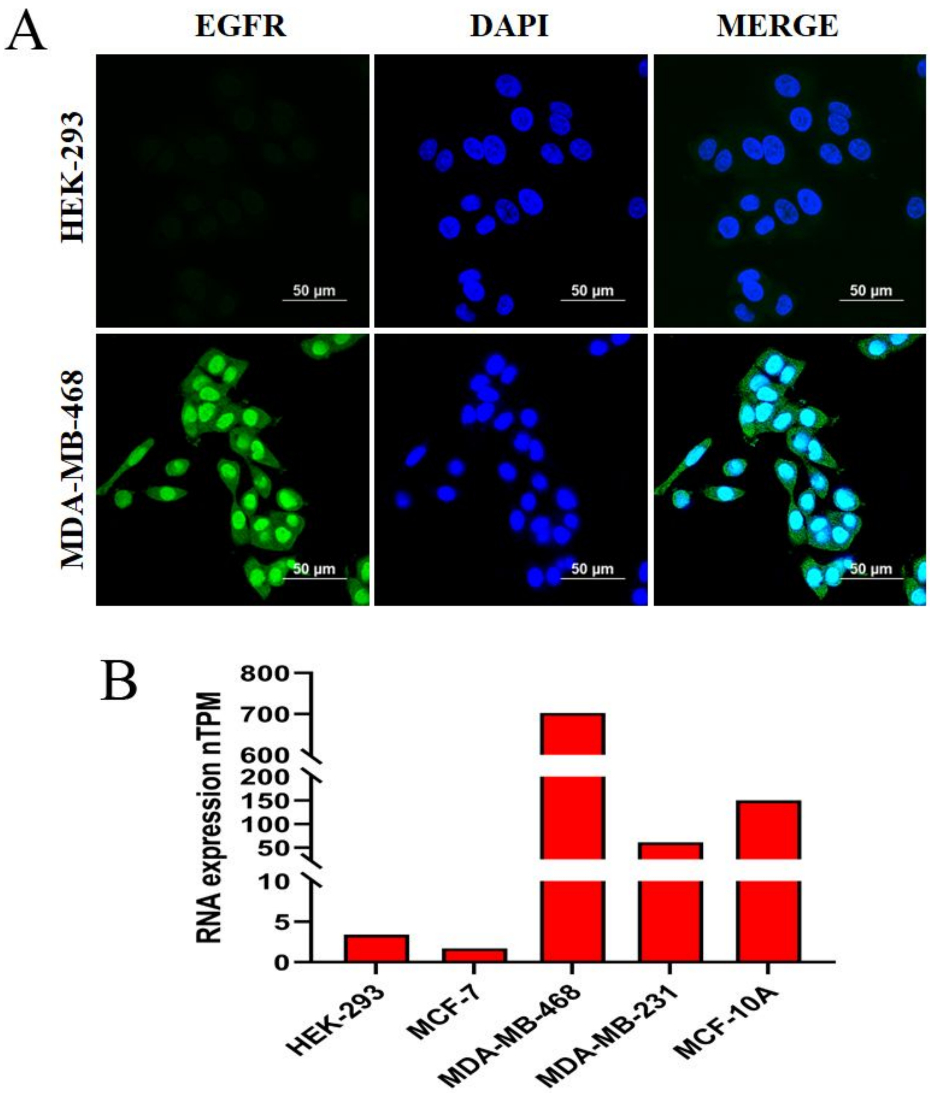

Figure S5: (A) EGFR expression was studied in MDA-MB-468 and HEK-293 cell lines, by immunofluorescence assay, (green fluorescence indicates EGFR expression, while blue represents a nuclear counterstain Hoechst 33342). MDA-MB-468 cells showed 75% increased expression of EGFR compared to HEK-293. The scale bar represents 50 µm. (B) EGFR transcription data as obtained from protein atlas database showing low EGFR transcription in case of non-cancerous HEK-293 and breast cancer MCF-7 cells, where very high transcription of EGFR is observed for MDA-MB-468 and mild EGFR transcription in MDA-MB-231. (Based on the data obtained from https://www.proteinatlas.org/ENSG00000146648-EGFR/cell+line#uncategorized).

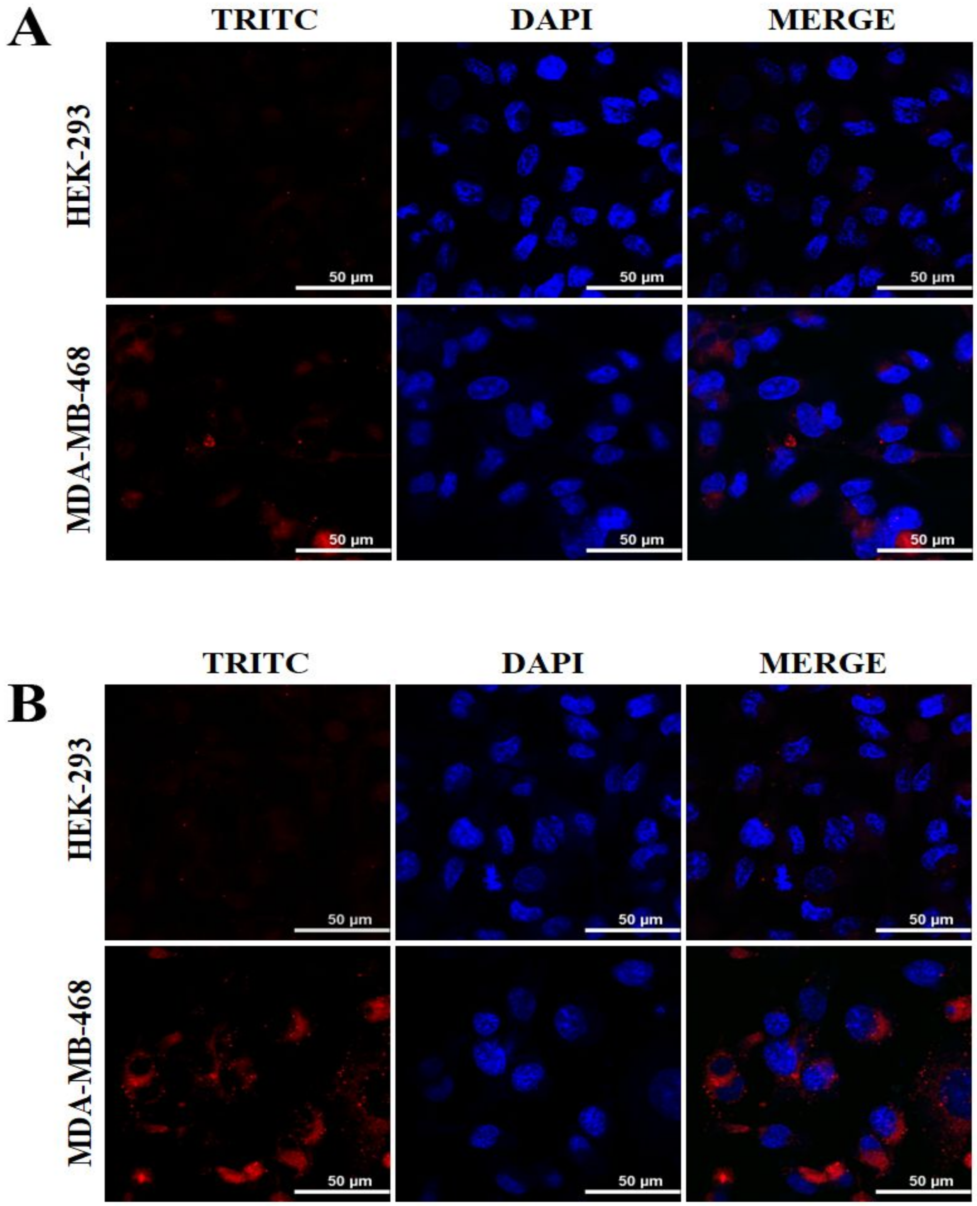

Figure S6: TRITC loaded cubosomes uptake was studied at (A) 12 h and (B) 24 h. MDA-MB-468 cells showed high uptake of cubosomes tagged with peptide, on the contrary HEK-293 cells have negligible uptake. This confirms selective uptake of peptide tagged cubosomes by EGFR expressing cells. The scale bar is 50 μm.

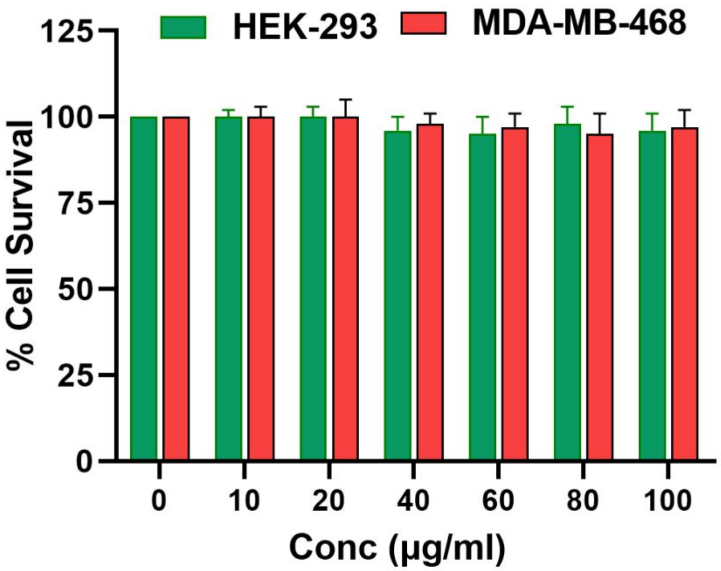

Figure S7: Cell survivability assay of bare cubosomes showing no significant toxicity in HEK-293 and MDA-MB-468 cell line when treated at 24 h.

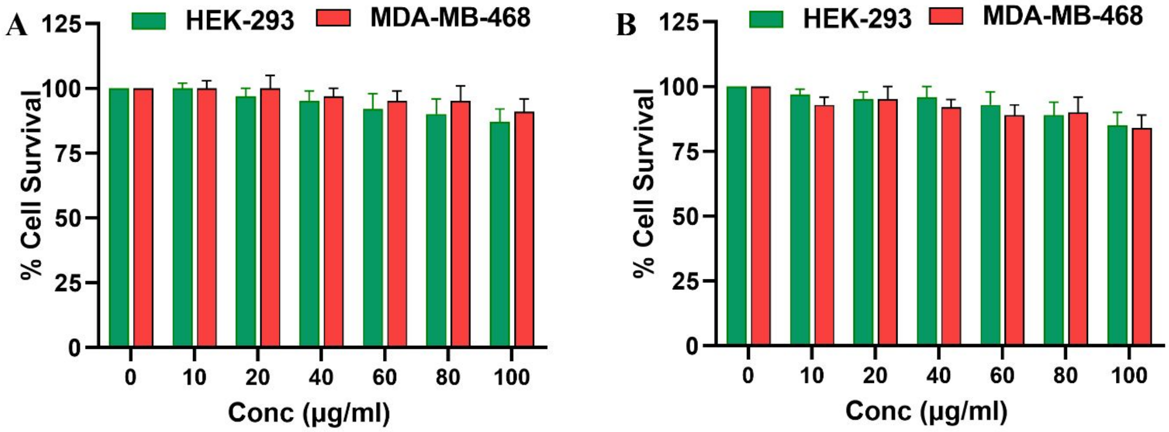

Figure S8: Cell survivability assay of paclitaxel loaded cubosomes (Cbs-Pxl) without peptide tagging (non-targeted delivery) for (A) 12 h and (B) 24 h. Cbs-Pxl did not show much toxicity in HEK-293 and MDA-MB-468 cells indicating negligible non-specific uptake of cubosome.

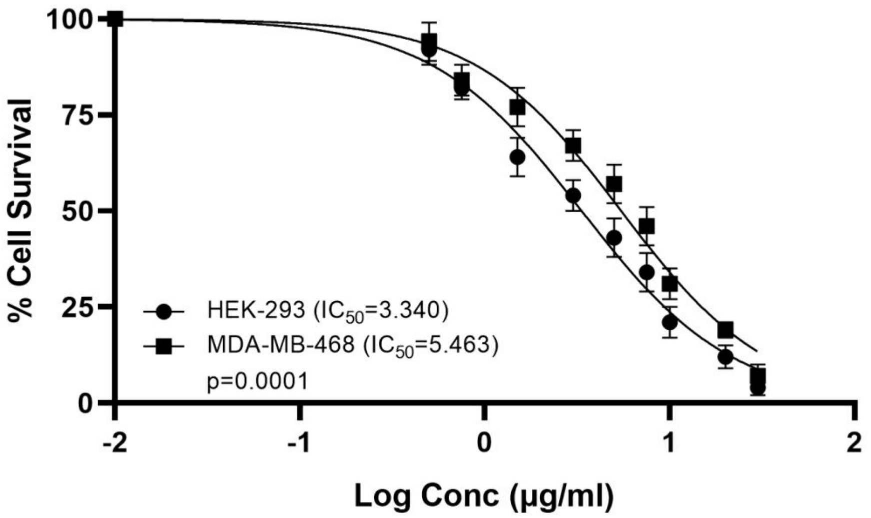

Figure S9: MTT assay of Paclitaxel on HEK-293 and MDA-MB-468 cells showing non-specific cytotoxicity of the drug at 24 h.

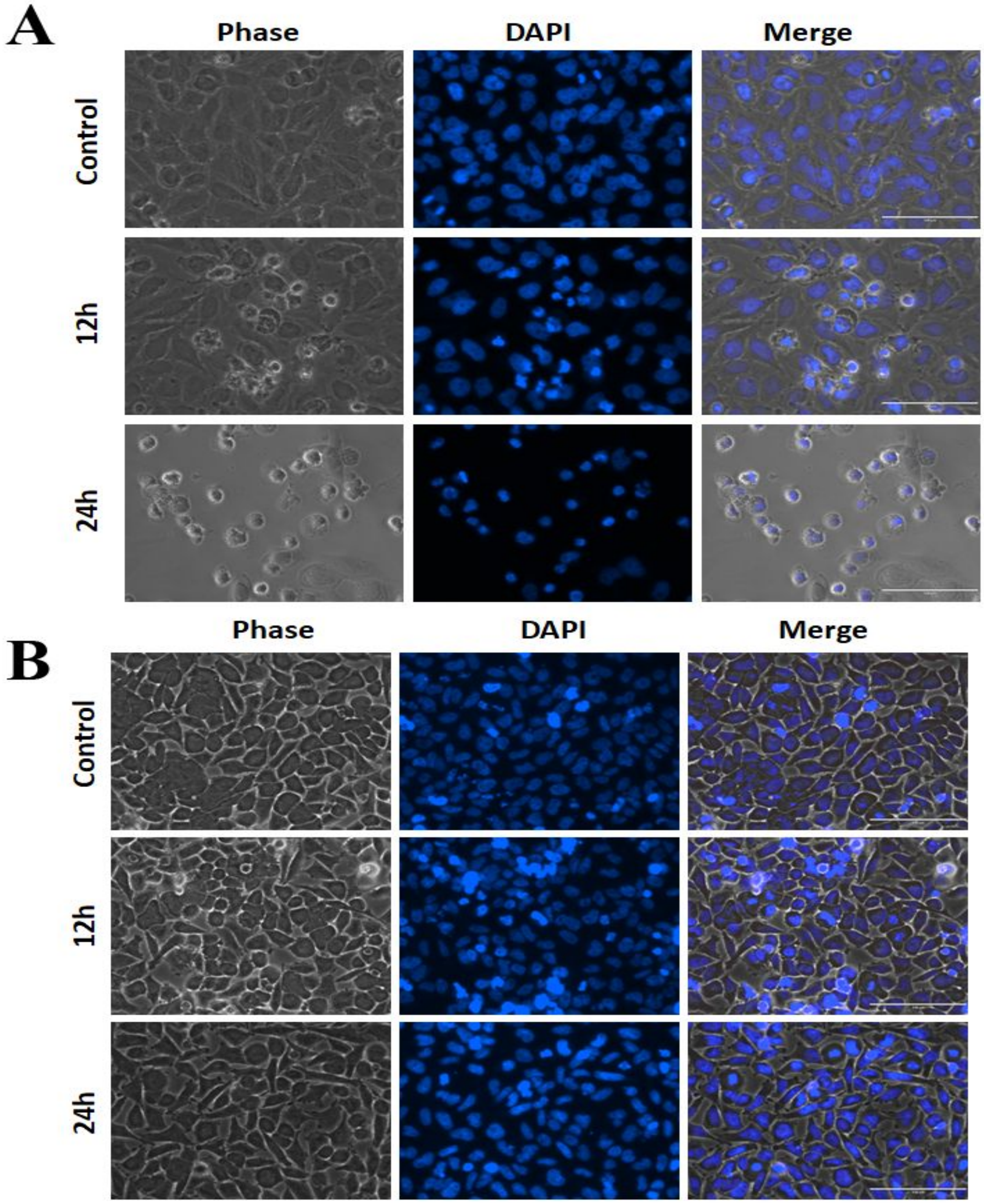

Figure S10: Cell and nuclear morphology study after treating with 53 µg/mL Cbs-Pxl-Pep shows morphological changes in (A) MDA-MB-468 indicating apoptosis induction; (B) HEK-293 cells showed no apoptotic morphology, indicating specificity of Cbs-Pxl-Pep to induce apoptosis in EGFR expressing MDA-MB-468. The scale bar represents 100 µm.

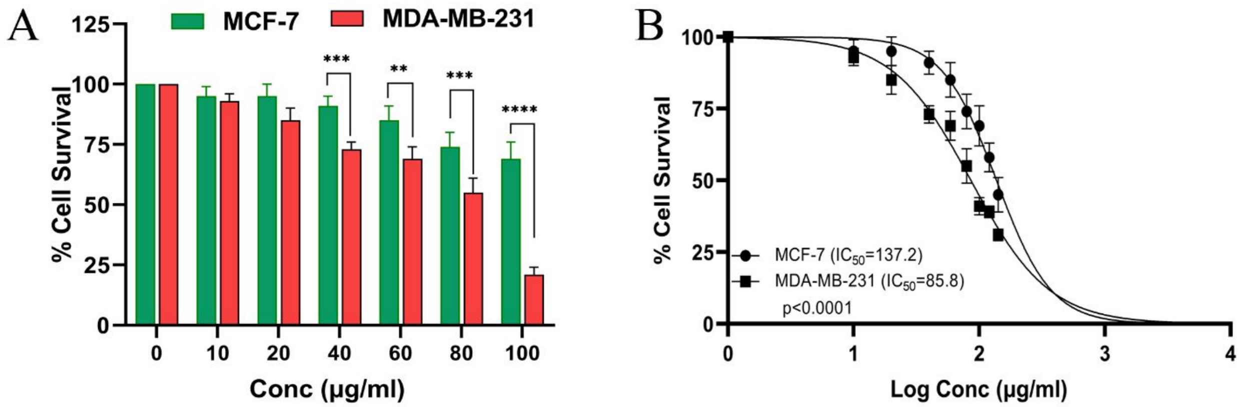

Figure S11. MTT data of Cbs-Pxl-Pep in EGFR+ve MDA-MB-231 and EGFR-ve MCF-7 cell lines at 24 h. (A) MDA-MB-231 cells showing dose dependent cytotoxicity for Cbs-Pxl-Pep whereas cytotoxicity observed in MCF-7 cells were lower. (B) Non-linear regression curve showing IC50 value of 137.2 µg/ml and 85.8 µg/ml in MCF-7 and MDA-MB-231 cells, respectively.

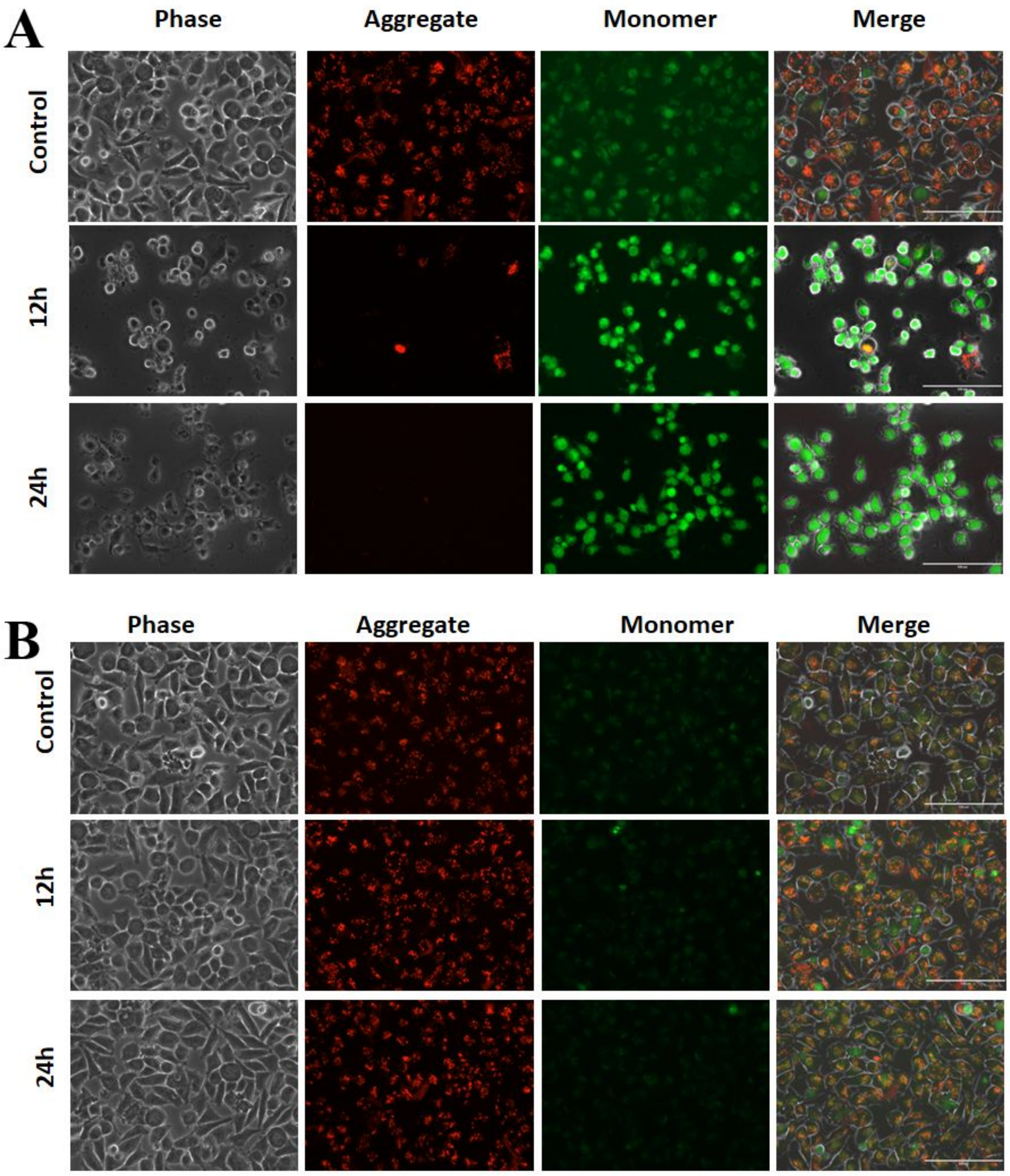

Figure S12: IC_{50} Cbs-Pxl-Pep induces mitochondrial membrane depolarization in (A) TNBC cell line MDA-MB-468, (B) but no significant change of mitochondrial membrane potential in HEK-293. This indicates specificity of Cbs-Pxl-Pep to induce mitochondrial depolarization in EGFR expressing MDA-MB-468 but not in EGFR negative HEK-293.

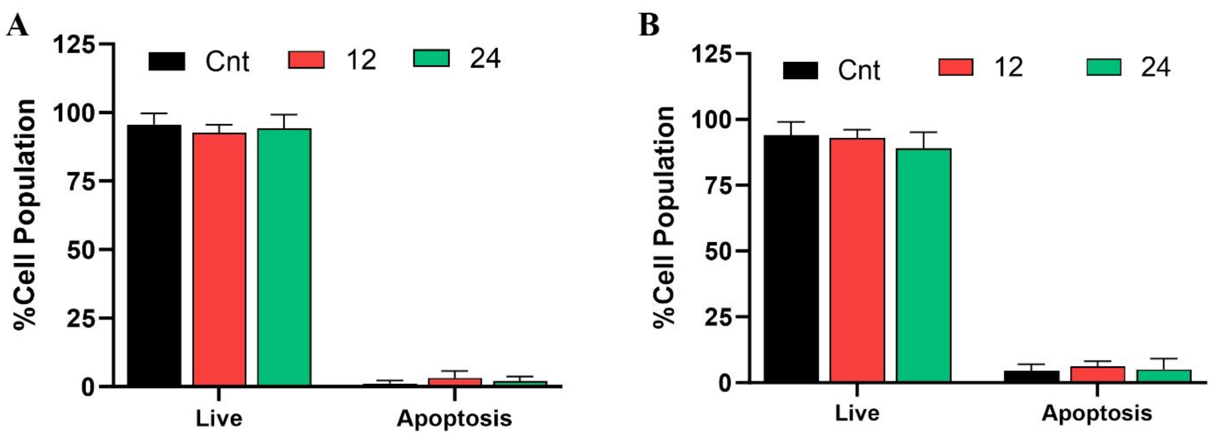

Figure S13: FACS analysis for apoptosis showing no significant change in (A) MDA-MB-468 and (B) HEK-293 cells before and after treatment with Cbs-Pxl. Thus indicating that without peptide tagging the drug loaded cubosome (Cbs-Pxl) is non-toxic in both the cells.
